# Physical activity prior to transient ischaemic attack and risk of subsequent cerebrovascular events: a Swedish register-based study

**DOI:** 10.1136/bmjopen-2026-124056

**Published:** 2026-09-21

**Authors:** Elīza Ance Beņislavska, Anastasios Mavridis, Malin Reinholdsson, Katharina S Sunnerhagen

**Affiliations:** 1 Institute of Neuroscience and Physiology, University of Gothenburg, Gothenburg, Sweden; 2 Riga Stradins University, Riga, Latvia; 3 Sahlgrenska Sjukhuset, Gothenburg, Sweden

**Keywords:** Stroke, Stroke Rehabilitation, STROKE MEDICINE, Physical Fitness, Neurology

## Abstract

**Objectives:**

To examine the association between pre-transient ischaemic attack (TIA) physical activity (PA) and subsequent cerebrovascular events.

**Design:**

Register-based cohort study

**Setting:**

Stroke units in Gothenburg, Sweden, using a local stroke register linked to national healthcare and demographic registers.

**Participants:**

1610 adults (51.7% female, mean age 72.5 years) with a registered TIA between November 2014 and December 2019.

**Primary and secondary outcome measures:**

The primary outcome was ischaemic stroke (International Classification of Diseases, 10th Revision I63 and I64). Secondary outcomes were total stroke (I61, I63 and I64) and a composite of stroke or TIA (I61, I63, I64 and G45.9). Time-to-event analyses were performed using Cox proportional hazards models with progressive adjustment for demographic, clinical and treatment-related factors.

**Results:**

Most participants reported light PA (50.4%). In crude analyses, moderate/vigorous PA was associated with lower hazards of ischaemic stroke (HR 0.36, 95% CI 0.16 to 0.78) and total stroke (HR 0.39, 95% CI 0.20 to 0.79) compared with sedentary individuals. These associations were attenuated after adjustment for age and sex and were no longer statistically significant (HR 0.48, 95% CI 0.22 to 1.08 for ischaemic stroke; HR 0.53, 95% CI 0.26 to 1.09 for total stroke). Further adjustment for clinical, socioeconomic and treatment-related factors did not materially alter the estimates.

**Conclusions:**

Higher pre-TIA PA was associated with substantially lower crude hazards of subsequent ischaemic and total stroke, but these associations were attenuated after accounting for age and remained similar with further adjustment. Pre-TIA PA may therefore reflect broader health and vascular risk rather than show a strong independent association with recurrence. However, imprecise estimates for moderate/vigorous PA remain compatible with a potentially lower stroke hazard and warrant further investigation.

**STRENGTHS AND LIMITATIONS OF THIS STUDY**

- Large, well-defined cohort identified through linked regional and national registries allowing adjustment for several sociodemographic, clinical and treatment-related factors with minimal loss to follow-up.
- Long-term follow-up, clinically relevant absolute risk estimates, and complementary Cox and competing-risk analyses enabled assessment of recurrence across different follow-up periods and strengthened the robustness of findings.
- Physical activity (PA) was assessed using a validated but relatively coarse self-reported ordinal measure and was therefore susceptible to social desirability and recall bias and potential misclassification. The relatively small size of the moderate/vigorous PA group may have reduced precision.
- Potential selection bias related to missing PA data, reverse causality and residual confounding by functional, behavioural and vascular characteristics not captured in the registers cannot be excluded.

## Background

 Transient ischaemic attack (TIA) is a brief episode of cerebral ischaemia without acute infarction, caused by temporary disruption of arterial blood flow to the brain, resulting in focal neurologic deficits that resolve after spontaneous restoration of blood flow.^1^ In the Framingham Heart Study, the incidence of TIA was estimated at 1.19 per 1000 person-years.^2^ In comparison, a Swedish population-based study using data from 2011 to 2012 reported a crude incidence of first-ever TIA of 0.74 per 1000 person-years.^3^

It is predicted that one in four adults will experience a stroke in their lifetime.^4^ TIA is a major predictor of future stroke, with individuals who have experienced a TIA having a 4.37-fold higher long-term risk of subsequent stroke.^2^ The 90-day risk of stroke after a TIA is approximately 2.7%.^5^ Stroke risk can be reduced through regular physical activity (PA) in individuals of both sexes and across all age groups.^6^ PA helps improve several modifiable stroke risk factors, such as hypertension, dyslipidaemia, diabetes and obesity,^6^ and is also associated with a healthier lifestyle, including less sedentary behaviour, lower alcohol consumption and less smoking.^6^ According to the WHO 2020 guidelines, all adults should undertake at least 150 to 300 min of moderate intensity, or 75 to 150 min of vigorous intensity PA, or an equivalent combination per week.^7^ However, almost one-third (31%) of the world’s adult population is physically inactive.^8^ Physical inactivity is the second highest attributable risk factor for stroke and almost one third of first stroke events are associated with it.^9 10^

Recurrent TIA and stroke events are associated with greater disability and higher mortality, highlighting the importance of secondary prevention.^11–13^ Previous research has shown that regular PA prior to TIA is associated with a lower risk of long-term disability following the event.^14^ However, despite growing evidence that PA improves overall health and reduces the risk of recurrent events,^15^ PA levels following stroke consistently fall below recommended guidelines.^16^ In addition, evidence regarding pre-TIA PA levels and their association with subsequent cerebrovascular events remains limited.^15^

The primary aim of this study was to examine the association between pre-TIA PA levels and subsequent cerebrovascular events following TIA, with ischaemic stroke as the primary outcome and total stroke and stroke/TIA as secondary outcomes.

## Methods

### Study design and data sources

This study was a register-based cohort study, based on two Swedish national and regional stroke care quality registers (Riksstroke, Väststroke) and two public databases: The Swedish National Patient Register (NPR)^17^ and Statistics Sweden (SCB).^18^ Väststroke includes all individuals with stroke or TIA treated at the stroke units at Sahlgrenska University Hospital, a university hospital organisation comprising three hospital sites in the Gothenburg area. The data were linked with the national stroke register, Riksstroke, for the same patients. Together, Väststroke and Riksstroke provided information on patient characteristics, stroke-related variables and outcomes. Demographic data were collected from Statistics Sweden^18^ and NPR^17^ was used for the collection of data on comorbidities and stroke outcomes.

The databases were linked by SCB and the National Board of Health and Welfare using Swedish personal identity numbers. All datasets were pseudonymised, with personal identity numbers replaced by serial numbers and no identifiable information was accessible to the researchers.

### Patient and public involvement

Patients and members of the public were not involved in the design, conduct, reporting or dissemination plans of this research.

### Study population

**Figure 1:**
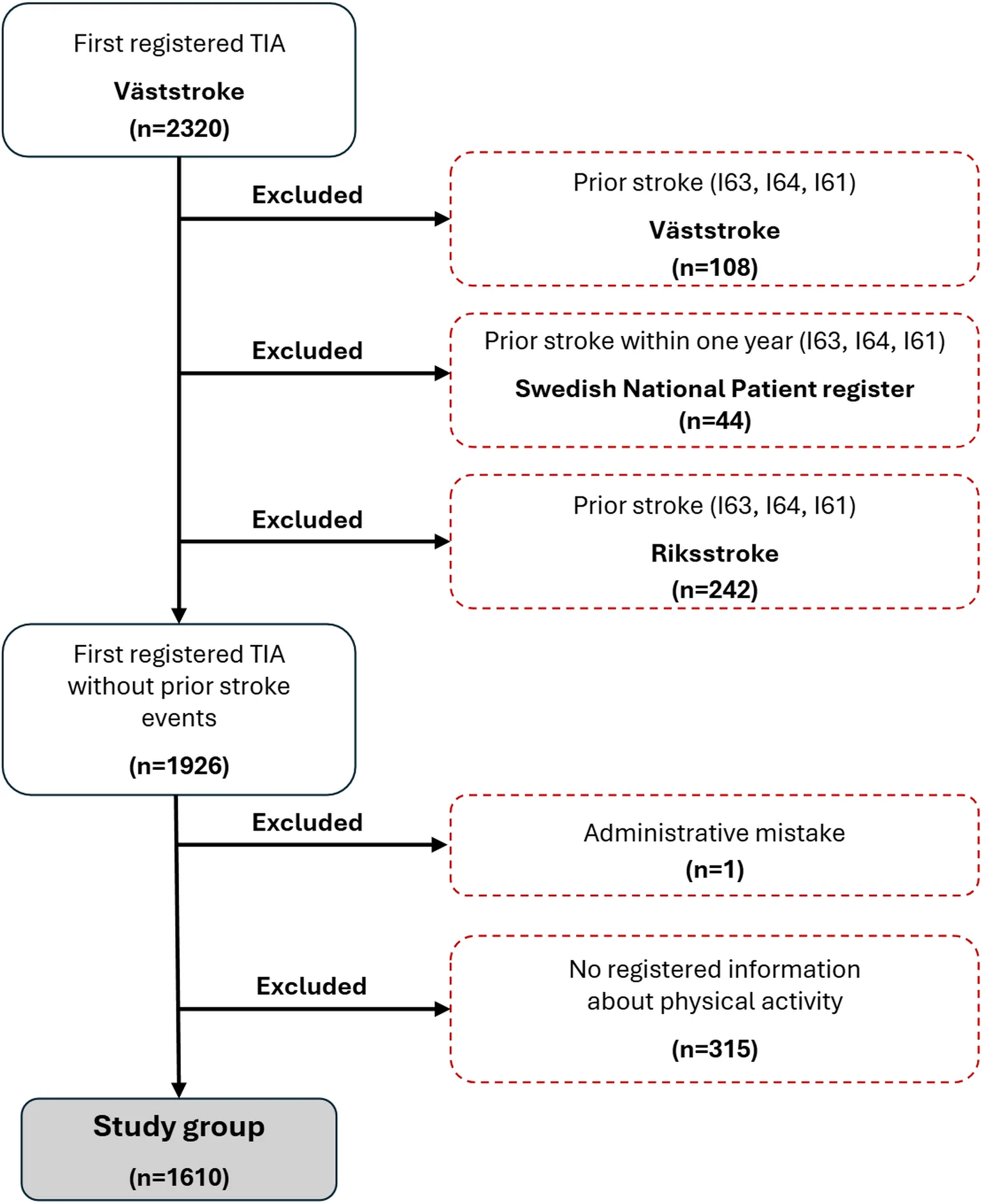
Flow chart of the study group selection. TIA, transient ischaemic attack.

The study population was identified through the local stroke and TIA register Väststroke between 1 November 2014 and 29 December 2019. Adult patients (≥18 years) with a TIA (International Classification of Diseases, 10th Revision (ICD-10) G45.9) registered in Väststroke during the study period were included, with the first registration during this period defined as the index event. Individuals were excluded if they had a previous diagnosis of ischaemic stroke (ICD-10 I63), unspecified stroke (ICD-10 I64) or haemorrhagic stroke (ICD-10 I61) recorded in Väststroke prior to the index date during the study period or in the NPR within 1 year prior to the index date. Individuals with missing information on PA prior to the first registered TIA were also excluded. One individual with a recorded date of death preceding the first TIA event was excluded as a probable administrative error (figure 1).

### Variables

#### Baseline characteristics

Information on age, sex, PA levels, smoking status, atrial fibrillation and medication use was collected from Riksstroke. Demographic data collected from Statistics Sweden encompassed country of birth, marital and employment status, education level and date of death. Country of birth was categorised as Sweden, EU countries excluding Sweden and rest of the world. Marital status was grouped as married/registered partner or never married/divorced/widowed. Employment status was categorised as employed or unemployed. Education level was divided into categories based on total years of studies: lower education (≤9 (10) years, according to the education system throughout the years), secondary education (10–11 years) and higher education (≥12 years). Information on pre-TIA medication use was obtained from Riksstroke and included antihypertensive, lipid-lowering, antiplatelet and anticoagulant therapies.

#### Comorbidities

Comorbidity burden was measured using the Swedish-adapted Charlson Comorbidity Index (CCI) developed by Ludvigsson *et al*^19^ including the following comorbidities: myocardial infarction, congestive heart failure, peripheral vascular disease, cerebrovascular disease, chronic obstructive pulmonary disease, other chronic pulmonary disease, rheumatic disease, dementia, hemiplegia or tetraplegia, diabetes with end organ damage, mild liver disease, moderate or severe liver disease, any malignancy including leukaemia and lymphoma, metastatic cancer and HIV/AIDS. Comorbidities were identified using ICD-10 and were assessed within 1 year prior to the first registered TIA event. Peptic ulcer disease was excluded due to insufficient coding detail. Kidney disease was also excluded, as it was not possible to distinguish chronic (N18) from acute (N17) kidney failure in the available data. Each comorbidity included was assigned a weight, and these were summed to obtain the total comorbidity score.

#### PA assessment

The Saltin-Grimby Physical Activity Level Scale (SGPALS) was used to assess PA prior to the index TIA and subsequent Väststroke registrations. It is a four-level ordinal scale used to assess self-reported leisure-time PA for clinical and research purposes and has demonstrated good concurrent validity against objective measures of PA and aerobic capacity, as well as good predictive validity for cardiovascular risk factors, morbidity and mortality.^20 21^ The levels are defined as:

1=Sedentary. Being almost completely inactive: reading, TV watching, movies, using computers or doing other sedentary activities during leisure time; 2=Some PA during at least 4 hours/week (riding a bicycle or walking to work, walking or skiing with the family, gardening, fishing, table tennis, bowling, etc); 3=Regular PA and training (moderate PA) such as heavy gardening, running, swimming, callisthenics, tennis, badminton and similar activities for at least 2–3 hours/week 4=Regular hard physical training for competition sports (vigorous PA): running events, orienteering, skiing, swimming, soccer, racing, European handball, etc several times per week.

PA was assessed in a standardised manner at the first encounter with a physiotherapist at the stroke unit. Participants were asked to estimate their average leisure-time PA before the event, generally referring to approximately the preceding year, with follow-up questions regarding intensity, duration and type of activity. Relatives could assist in confirming the reported PA level when needed.

Levels 3 and 4 were combined into a single category due to the small number of individuals in level 4. Throughout the text, the PA levels are referred to as follows: level 1—sedentary, level 2—light PA and levels 3–4 moderate/vigorous PA.

#### Outcomes

The primary outcome was ischaemic stroke (ICD-10 I63 and I64). Secondary outcomes were total stroke (I61, I63 and I64) and a composite outcome of stroke or TIA (I61, I63, I64 and G45.9). Unspecified stroke (I64) was classified as ischaemic stroke, as brain CT is routinely performed in Swedish stroke care (98% of patients with ischaemic stroke in the 2019 Riksstroke report),^22^ making it unlikely that an intracerebral haemorrhage would remain unspecified. Furthermore, unspecified stroke represented only approximately 1% of all stroke registrations in Riksstroke.^22^ TIA was defined as G45.9. Outcomes were collected from both the NPR and Väststroke up to 31 December 2021.

Time to event was calculated from the first registered TIA until the first occurrence of the respective outcome. Individuals were censored at death or at the end of follow-up. For each outcome, only the first event during follow-up was considered.

### Statistics

#### Missing data analysis

Participants with available PA data were compared with those with missing PA data to assess potential selection associated with missing exposure information. Continuous variables were compared using the Mann-Whitney U test, while categorical demographic, socioeconomic, clinical and treatment characteristics were compared using Pearson’s χ^2^ test.

#### Descriptive characteristics

Descriptive characteristics of the study sample were summarised using means, SD and ranges for continuous variables, and counts and percentages for categorical variables. Descriptive characteristics were reported for the total sample and stratified by baseline PA level.

#### Cumulative incidence

Cumulative incidence of ischaemic stroke, total stroke and stroke or TIA was estimated across PA levels using the Aalen-Johansen estimator, with death treated as a competing event. Cumulative incidence functions are presented with 95% CIs and numbers at risk. Absolute cumulative incidence estimates with 95% CIs were additionally calculated at 7, 30 and 90 days and at 1, 3 and 5 years after the index TIA.

#### Hazard of subsequent events

HRs for the association between PA levels prior to first registered TIA and time to the first recurrent event were estimated using Cox proportional hazards (PH) regression models. Separate models were fitted for each outcome: ischaemic stroke (I63, I64), total stroke (I61, I63, I64) and stroke or TIA (I61, I63, I64, G45.9). The PH assumption was evaluated using Schoenfeld residuals, while multicollinearity among covariates was assessed using the variance inflation factor (VIF) with a cut-off value of 2.5 to ensure that no covariates exhibited problematic collinearity.^23^ For all models, variables seemed to fulfil the PH assumptions. Given the exploratory observational design, sequential PH models were fitted to examine how the association between PA and subsequent events changed with progressive adjustment. Model 1 included PA only. Model 2 additionally included age and sex because of their established relationships with both PA and cerebrovascular risk. Model 3 further included CCI to capture overall comorbidity burden, atrial fibrillation as an established stroke risk factor, and education and country of birth as indicators of socioeconomic circumstances. Model 4 additionally included antihypertensive, lipid-lowering, antiplatelet and anticoagulant medication use to account for differences in cardiovascular treatment and underlying vascular risk. All models were fitted separately for each outcome using complete-case analysis for the covariates included in each model. Consequently, participants with missing covariate information were excluded from models 3 and 4, resulting in 1568 participants in these models compared with 1610 in models 1 and 2.

#### Competing-risk regression

To account for death as a competing event, Fine-Gray regression was additionally performed for each outcome to estimate subdistribution HRs (sHRs) with 95% CIs. The same four sequential adjustment models as in the Cox regression analyses were applied.

#### Sensitivity analyses

Age distributions across PA groups were first examined using density plots to assess the degree of overlap. PA×age interaction and potential non-linearity of age were subsequently assessed in both the age- and sex-adjusted and fully adjusted models. Interaction was evaluated using likelihood-ratio tests comparing models with and without PA×age interaction terms, while non-linearity was assessed by comparing linear age with a natural cubic spline with four df. Finally, Cox models were repeated among participants aged 60–79 years, where substantial age overlap was present across all PA groups.

#### PA changes

As an exploratory descriptive analysis, Sankey diagrams were used to illustrate changes in PA levels across recurrent events/no events. A multicolumn layout was applied, where each column represented either PA level or event type (baseline PA, PA prior to the first recurrent event, first recurrent event type, PA prior to the second recurrent event and second recurrent event type). Repeat PA measurements were available only at subsequent Väststroke registrations and, consequently, the analysis was restricted to participants with recurrent registrations.

All statistical tests were 2-tailed and performed at α 5%. Given the exploratory and hypothesis-generating nature of the study, no adjustment for multiple comparisons was applied.^24^ P values were interpreted alongside effect estimates and 95% CIs, and findings from secondary covariates should be interpreted cautiously. Statistical analyses were performed using R (R Core Team (2023), R: A Language and Environment for Statistical Computing, R Foundation for Statistical Computing, Vienna, Austria) and SPSS (IBM SPSS Statistics for Windows, V.29.0, released 2022; IBM).

## Results

### Study population

There were 2320 individuals with a first TIA registered in Väststroke during the study period. Of those, 394 were excluded due to previous stroke diagnoses, and 315 were excluded due to missing data on PA. One patient was additionally excluded due to administrative errors. The final study sample consisted of 1610 individuals (figure 1). Median follow-up was 3.89 years (IQR 2.63–5.39) for ischaemic stroke, corresponding to 6329 person-years. Corresponding follow-up was 3.88 years (IQR 2.62–5.37; 6316 person-years) for total stroke and 3.34 years (IQR 2.08–5.11; 5462 person-years) for stroke/TIA. During follow-up, 124 participants experienced ischaemic stroke, 137 experienced total stroke (including 13 haemorrhagic strokes), and 379 experienced stroke or TIA.

### Missing data analysis

Participants with and without available PA data were similar across demographic, socioeconomic and clinical characteristics, including age, sex, comorbidity, atrial fibrillation, smoking, education, marital status, employment status, country of birth, antiplatelet use and anticoagulant use (online supplemental table 1). Participants with missing PA data were less frequently treated with antihypertensive medication (49% vs 56%, p=0.021) and lipid-lowering medication (18% vs 27%, p=0.001).

### Descriptive characteristics

The study sample consisted of 1610 individuals of whom 48.3% were male (n=777) and 51.7% female (n=833). Age ranged from 27 to 102 years, with a mean (SD) age of 72.5 (11.7) years (median (IQR)=73 (16)). Descriptive characteristics of the study population stratified by PA levels are shown in table 1.

**Table 1:** Descriptive characteristics of the study population stratified by PA levels

Physical activity level prior TIA	Sedentary, n (valid %)	Light PA, n (valid %)	Moderate/vigorous PA, n (valid %)	Total, n (valid %)
	606 (37.6)	811 (50.4)	193 (12.0)	1610 (100)
Age^1^ at event, mean (SD)	75.0 (12.5)	72.1 (10.7)	66.9 (11.0)	72.5 (11.7)
Sex^2^				
Male	241 (39.8)	410 (50.6)	126 (65.3)	777 (48.3)
Female	365 (60.2)	401 (49.4)	67 (34.7)	833 (51.7)
Smoking^3^	64 (11.9)	58 (7.9)	9 (4.9)	131 (9.0)
CCI,^4^ mean (SD)	0.26 (0.8)	0.12 (0.5)	0.10 (0.4)	0.17 (0.6)
Atrial fibrillation^5^	141 (23.3)	129 (15.9)	20 (10.4)	290 (18.0)
Education level^6^				
Lower	186 (32.4)	217 (27.1)	24 (12.4)	427 (27.2)
Secondary	245 (42.7)	360 (44.9)	88 (45.6)	693 (44.2)
Higher	143 (24.9)	224 (28.0)	81 (42.0)	448 (28.6)
Marital status^7^				
Married/registered partner	265 (45.4)	433 (53.8)	118 (61.1)	816 (51.6)
Never married/divorced/widowed	319 (54.6)	372 (46.2)	75 (38.9)	766 (48.4)
Employment status^8^				
Employed	112 (19.2)	198 (24.6)	88 (45.6)	398 (25.2)
Unemployed	472 (80.8)	607 (75.4)	105 (54.4)	1184 (74.8)
Country of birth^9^				
Sweden	489 (80.7)	683 (84.2)	169 (87.6)	1341 (83.3)
EU without Sweden	91 (15.0)	89 (11.0)	16 (8.3)	196 (12.2)
Rest of the world	26 (4.3)	39 (4.8)	8 (4.1)	73 (4.5)
Use of medication				
Antihypertensives^10^	388 (64.0)	445 (54.9)	76 (39.4)	909 (56.5)
Lipid-lowering medication^11^	176 (29.0)	207 (25.5)	44 (22.8)	427 (26.5)
Antiplatelets^12^	141 (23.3)	160 (19.7)	34 (17.6)	335 (20.8)
Anticoagulants^13^	100 (16.5)	78 (9.6)	9 (4.7)	187 (11.6)

Missing values 1:0, 2:0, 3:154, 4:0. 5:0, 6:42, 7:28, 8:28, 9:0, 10:0, 11:0, 12:0, 13:0.

CCI, Charlson Comorbidity Index; EU, European Union; PA, physical activity; TIA, transient ischaemic attack.

Half (50.4%) of the study population reported light PA. Sedentary individuals were progressively older (mean age=75.0, SD=12.5), compared with moderate/vigorous PA group (mean age=66.9, SD=11.0). A larger proportion of participants were female in the lowest PA group (60.2% females) compared with the moderate/vigorous PA level (34.7% females). CCI was slightly lower among more active individuals and atrial fibrillation occurred more frequently among sedentary individuals.

A larger proportion of individuals with moderate/vigorous PA had a higher education level compared with the sedentary or light PA group. The proportion of partnered individuals, as well as employed individuals, was also higher in the moderate/vigorous PA group. The percentage of participants born in Sweden was slightly higher in the moderate/vigorous PA group.

Medication use varied across PA levels. Antihypertensives were the most commonly used medication in this study population, although their use was less frequent among individuals with moderate/vigorous PA levels. Similarly, lipid-lowering agents, antiplatelets and anticoagulants were less commonly used in the moderate/vigorous PA group.

### Cumulative incidence

**Figure 2:**
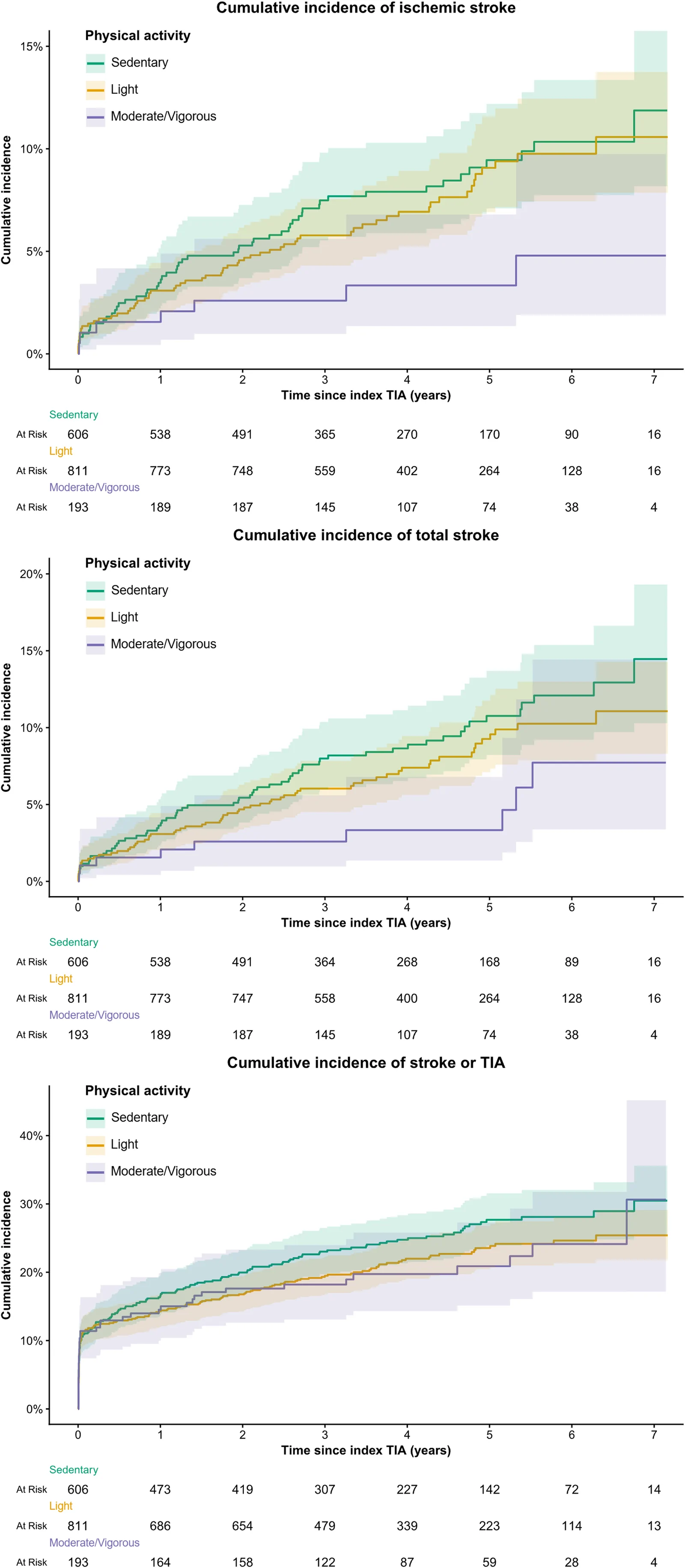
Aalen-Johansen cumulative incidence curves for cerebrovascular outcomes stratified by pre-TIA physical activity level, with death treated as a competing event. Shaded areas represent 95% CIs, and numbers at risk are presented below each panel. TIA, transient ischaemic attack.

Aalen-Johansen cumulative incidence curves accounting for death as a competing event are presented in figure 2. For ischaemic stroke, cumulative incidence during the first 90 days was similar across PA groups, reaching 1.5% (95% CI 0.7% to 2.7%) in sedentary participants, 1.6% (95% CI 0.9% to 2.7%) in those with light PA and 1.6% (95% CI 0.4% to 4.2%) in those with moderate/vigorous PA. During longer follow-up, the moderate/vigorous PA group showed greater separation from the sedentary and light PA groups, although CIs overlapped and estimates for the moderate/vigorous PA group were imprecise.

A similar pattern was observed for total stroke, with relatively low cumulative incidence early after the index TIA and greater separation of the moderate/vigorous PA group during longer follow-up. In contrast, the stroke/TIA composite showed a steep increase in cumulative incidence during the initial days following the index TIA across all PA groups, followed by a more gradual increase over time. Differences between PA groups were less pronounced for this composite outcome, with substantial overlap of the CIs. Absolute cumulative incidence estimates at 7, 30 and 90 days and at 1, 3 and 5 years are presented in table 2.

**Table 2:** Absolute cumulative incidence of cerebrovascular outcomes at prespecified follow-up time points by pre-TIA physical activity level, estimated using the Aalen-Johansen method with death as a competing event

Physical activity level	Time point					
	7 days	30 days	90 days	1 year	3 years	5 years
	Ischaemic stroke					
Sedentary	0.83% (0.32%, 1.8%)	0.99% (0.41%, 2.1%)	1.5% (0.74%, 2.7%)	3.5% (2.2%, 5.1%)	7.5% (5.5%, 9.8%)	9.4% (7.1%, 12%)
Light	0.99% (0.47%, 1.9%)	1.4% (0.72%, 2.3%)	1.6% (0.90%, 2.7%)	3.1% (2.0%, 4.4%)	5.8% (4.3%, 7.6%)	9.1% (6.9%, 12%)
Moderate/vigorous	0.52% (0.05%, 2.7%)	1.0% (0.21%, 3.4%)	1.6% (0.43%, 4.2%)	1.6% (0.43%, 4.2%)	2.6% (0.97%, 5.6%)	3.3% (1.4%, 6.8%)
	**Total stroke**					
Sedentary	0.83% (0.32%, 1.8%)	1.2% (0.52%, 2.3%)	1.7% (0.85%, 2.9%)	3.6% (2.3%, 5.3%)	8.0% (6.0%, 10%)	11% (8.2%, 14%)
Light	0.99% (0.47%, 1.9%)	1.4% (0.72%, 2.3%)	1.6% (0.90%, 2.7%)	3.1% (2.0%, 4.4%)	6.0% (4.5%, 7.8%)	9.3% (7.1%, 12%)
Moderate/vigorous	0.52% (0.05%, 2.7%)	1.0% (0.21%, 3.4%)	1.6% (0.43%, 4.2%)	1.6% (0.43%, 4.2%)	2.6% (0.97%, 5.6%)	3.3% (1.4%, 6.8%)
	**Stroke or TIA**					
Sedentary	8.9% (6.8%, 11%)	11% (8.7%, 14%)	13% (10%, 15%)	17% (14%, 20%)	23% (20%, 26%)	28% (24%, 32%)
Light	9.4% (7.5%, 12%)	11% (9.4%, 14%)	12% (10%, 15%)	14% (12%, 17%)	19% (17%, 22%)	24% (20%, 27%)
Moderate/vigorous	10% (6.6%, 15%)	11% (7.4%, 16%)	12% (7.8%, 17%)	15% (10%, 20%)	18% (13%, 24%)	21% (15%, 27%)

Data are presented as cumulative incidence estimates (95% confidence intervals).

TIA, transient ischaemic attack.

### Hazard of recurrent events

The results of the Cox proportional hazards regression models for ischaemic stroke, total stroke and the composite outcome of stroke or TIA are presented in tables 3–5.

**Table 3:** Cox proportional hazards models with varying adjustments for ischaemic stroke

	Model 1		Model 2		Model 3		Model 4	
	HR (95% CI)	P value	HR (95% CI)	P value	HR (95% CI)	P value	HR (95% CI)	P value
Physical activity (Ref.: Sedentary)								
Light	0.81 (0.56 to 1.16)	0.252	0.90 (0.62 to 1.30)	0.572	0.92 (0.63 to 1.35)	0.663	0.93 (0.63 to 1.36)	0.699
Moderate/vigorous	0.36 (0.16 to 0.78)	0.010	0.48 (0.22 to 1.08)	0.077	0.49 (0.22 to 1.11)	0.086	0.52 (0.23 to 1.16)	0.111
Age	NA	NA	1.05 (1.03 to 1.07)	<0.001	1.05 (1.03 to 1.07)	<0.001	1.04 (1.02 to 1.06)	<0.001
Sex (Ref.: Male)	NA	NA	0.93 (0.65 to 1.35)	0.718	0.94 (0.65 to 1.38)	0.764	0.97 (0.66 to 1.41)	0.865
CCI	NA	NA	NA	NA	0.89 (0.63 to 1.25)	0.501	0.86 (0.59 to 1.23)	0.399
Atrial fibrillation	NA	NA	NA	NA	1.03 (0.66 to 1.62)	0.889	0.90 (0.48 to 1.66)	0.730
Education (Ref.: Lower)								
Secondary	NA	NA	NA	NA	1.18 (0.76 to 1.81)	0.460	1.18 (0.76 to 1.81)	0.457
Higher	NA	NA	NA	NA	1.05 (0.63 to 1.72)	0.862	1.04 (0.63 to 1.71)	0.879
Country of birth (Ref.: Sweden)								
EU without Sweden	NA	NA	NA	NA	0.90 (0.52 to 1.58)	0.723	0.90 (0.52 to 1.58)	0.722
Rest of the world	NA	NA	NA	NA	0.84 (0.26 to 2.70)	0.774	0.78 (0.24 to 2.51)	0.679
Use of medication								
Antihypertensives (Ref.: No)	NA	NA	NA	NA	NA	NA	1.51 (0.99 to 2.31)	0.056
Lipid-lowering medication (Ref.: No)	NA	NA	NA	NA	NA	NA	0.81 (0.52 to 1.25)	0.342
Antiplatelets (Ref.: No)	NA	NA	NA	NA	NA	NA	1.50 (0.97 to 2.34)	0.071
Anticoagulants (Ref.: No)	NA	NA	NA	NA	NA	NA	1.22 (0.58 to 2.56)	0.607
Total cases, events	Total 1610, events 124				Total 1568, events 119			

CCI, Charlson Comorbidity Index; EU, European Union; NA, not applicable; Ref, reference.

**Table 4:** Cox proportional hazards models with varying adjustments for total stroke

	Model 1		Model 2		Model 3		Model 4	
	HR (95% CI)	P value	HR (95% CI)	P value	HR (95% CI)	P value	HR (95% CI)	P value
Physical activity (Ref.: Sedentary)								
Light	0.73 (0.52 to 1.04)	0.080	0.81 (0.57 to 1.15)	0.245	0.82 (0.57 to 1.18)	0.282	0.82 (0.57 to 1.18)	0.280
Moderate/vigorous	0.39 (0.20 to 0.79)	0.009	0.53 (0.26 to 1.09)	0.083	0.54 (0.26 to 1.11)	0.094	0.55 (0.27 to 1.14)	0.109
Age	NA	NA	1.05 (1.03 to 1.07)	<0.001	1.05 (1.03 to 1.07)	<0.001	1.04 (1.02 to 1.06)	<0.001
Sex (Ref.: Male)	NA	NA	0.92 (0.65 to 1.30)	0.628	0.92 (0.64 to 1.31)	0.637	0.94 (0.66 to 1.35)	0.748
Comorbidity index	NA	NA	NA	NA	0.83 (0.59 to 1.19)	0.317	0.80 (0.55 to 1.16)	0.240
Atrial fibrillation	NA	NA	NA	NA	1.06 (0.69 to 1.62)	0.803	1.06 (0.60 to 1.85)	0.849
Education (Ref.: Lower)								
Secondary	NA	NA	NA	NA	1.01 (0.67 to 1.52)	0.957	1.01 (0.67 to 1.52)	0.957
Higher	NA	NA	NA	NA	1.00 (0.63 to 1.59)	0.986	0.99 (0.62 to 1.58)	0.981
Country of birth (Ref.: Sweden)								
EU without Sweden	NA	NA	NA	NA	0.94 (0.56 to 1.59)	0.824	0.95 (0.56 to 1.61)	0.854
Rest of the world	NA	NA	NA	NA	1.07 (0.39 to 2.95)	0.893	0.98 (0.36 to 2.72)	0.976
Use of medication								
Antihypertensives (Ref.: No)	NA	NA	NA	NA	NA	NA	1.34 (0.90 to 1.99)	0.151
Lipid-lowering medication (Ref.: No)	NA	NA	NA	NA	NA	NA	0.84 (0.56 to 1.28)	0.426
Antiplatelets (Ref.: No)	NA	NA	NA	NA	NA	NA	1.59 (1.05 to 2.42)	0.028
Anticoagulants (Ref.: No)	NA	NA	NA	NA	NA	NA	1.00 (0.49 to 2.03)	0.998
Total cases, events	Total 1610, events 137				1568, events 132			

CCI, Charlson Comorbidity Index; EU, European Union; NA, not applicable; Ref, reference.

**Table 5:** Cox proportional hazards models with varying adjustments for stroke or TIA

	Model 1		Model 2		Model 3		Model 4	
	HR (95% CI)	P value	HR (95% CI)	P value	HR (95% CI)	P value	HR (95% CI)	P value
Physical activity (Ref.: Sedentary)								
Light	0.80 (0.64 to 0.99)	0.039	0.86 (0.69 to 1.07)	0.174	0.90 (0.72 to 1.13)	0.373	0.91 (0.73 to 1.14)	0.426
Moderate/vigorous	0.76 (0.54 to 1.07)	0.119	0.93 (0.65 to 1.32)	0.667	1.01 (0.70 to 1.45)	0.958	1.03 (0.72 to 1.48)	0.868
Age	NA	NA	1.02 (1.01 to 1.03)	<0.001	1.02 (1.01 to 1.03)	<0.001	1.02 (1.01 to 1.03)	0.004
Sex (Ref.: Male)	NA	NA	1.22 (0.99 to 1.51)	0.062	1.26 (1.02 to 1.57)	0.035	1.30 (1.04 to 1.61)	0.019
Comorbidity index	NA	NA	NA	NA	0.97 (0.81 to 1.16)	0.761	0.95 (0.79 to 1.15)	0.618
Atrial fibrillation	NA	NA	NA	NA	1.13 (0.86 to 1.47)	0.373	1.03 (0.71 to 1.48)	0.891
Education (Ref.: Lower)								
Secondary	NA	NA	NA	NA	0.98 (0.76 to 1.26)	0.875	0.98 (0.76 to 1.25)	0.852
Higher	NA	NA	NA	NA	0.87 (0.65 to 1.15)	0.323	0.87 (0.66 to 1.16)	0.340
Country of birth (Ref.: Sweden)								
EU without Sweden	NA	NA	NA	NA	1.05 (0.77 to 1.43)	0.767	1.05 (0.77 to 1.44)	0.747
Rest of the world	NA	NA	NA	NA	1.50 (0.92 to 2.45)	0.102	1.48 (0.91 to 2.43)	0.115
Use of medication								
Antihypertensives (Ref.: No)	NA	NA	NA	NA	NA	NA	1.05 (0.83 to 1.33)	0.677
Lipid-lowering medication (Ref.: No)	NA	NA	NA	NA	NA	NA	1.07 (0.84 to 1.37)	0.591
Antiplatelets (Ref.: No)	NA	NA	NA	NA	NA	NA	1.26 (0.96 to 1.64)	0.096
Anticoagulants (Ref.: No)	NA	NA	NA	NA	NA	NA	1.22 (0.79 to 1.89)	0.373
Total cases, events	Total 1610, events 379				Total 1568, events 364			

CCI, Charlson Comorbidity Index; EU, European Union; NA, not applicable; Ref, reference.

In crude Cox regression analyses, individuals with moderate/vigorous PA had 64% lower hazard for ischaemic stroke (HR=0.36, 95% CI 0.16 to 0.78) and 61% lower hazard for total stroke (HR=0.39, 95% CI 0.20 to 0.79) compared to sedentary individuals. No statistically significant difference was found between individuals with light PA and sedentary for neither ischaemic nor total stroke. For the stroke or TIA outcome, Cox regression analysis showed that compared with sedentary individuals, those with light PA had 20% lower hazard for stroke or TIA (HR=0.80, 95% CI 0.64 to 0.99), but moderate/vigorous PA did not reach statistical significance.

After adjusting for age and sex, there was no statistically significant association between PA and any of the outcomes. Age was significantly associated with an increased hazard of all outcomes, with each additional year corresponding to a 5% higher hazard for ischaemic stroke (HR=1.05, 95% CI 1.03 to 1.07) and total stroke (HR=1.05, 95% CI 1.03 to 1.07) and 2% higher hazard for stroke/TIA (HR=1.02, 95% CI 1.01 to 1.03).

In model 4, PA was not statistically significantly associated with any outcome. Age remained significantly associated with increased hazard across outcomes, and antiplatelet use was associated with higher hazard of total stroke (HR=1.59, 95% CI 1.05 to 2.42). Female sex was associated with a higher hazard of stroke or TIA (HR=1.30, 95% CI 1.04 to 1.61). Similar patterns were observed in model 3 that excluded medication use.

### Competing-risk regression

Fine-Gray regression accounting for death as a competing event yielded results consistent with the Cox analyses (online supplemental tables 2-4). For ischaemic stroke, moderate/vigorous PA was associated with lower subdistribution hazard in the crude model (sHR 0.41, 95% CI 0.19 to 0.90). The association was attenuated after adjustment for age and sex (sHR 0.56, 95% CI 0.25 to 1.23) and remained similar in the fully adjusted model (sHR 0.57, 95% CI 0.25 to 1.28). Similar attenuation after adjustment was observed for total stroke, while no association was observed for the stroke/TIA composite after adjustment.

### Sensitivity analyses

Age distributions differed across PA groups but showed substantial overlap (online supplemental figure 1). There was no evidence of interaction between PA and age in either the age- and sex-adjusted model (P for interaction=0.689) or the fully adjusted model (P for interaction=0.670). Similarly, there was no evidence of non-linearity in the association between age and ischaemic stroke in either model (P for non-linearity=0.536 and 0.368, respectively). In analyses restricted to participants aged 60–79 years, PA estimates remained similar to those in the full cohort. The age- and sex-adjusted HRs were 0.81 (95% CI 0.47 to 1.41) for light PA and 0.52 (95% CI 0.19 to 1.38) for moderate/vigorous PA. Corresponding fully adjusted HRs were 0.80 (95% CI 0.45 to 1.42) and 0.57 (95% CI 0.21 to 1.56), respectively.

### Changes in PA levels and subsequent event outcomes

An exploratory descriptive analysis of PA transitions among participants with recurrent Väststroke registrations is presented in (online supplemental figure 2). Most participants remained at the same PA level between registrations. Descriptively, stroke events were more frequently observed among participants who remained sedentary or decreased their PA, whereas TIA events were more frequently observed among those with higher or increasing PA levels. These patterns should be interpreted cautiously because repeat PA measurements were conditional on recurrent Väststroke registration.

## Discussion

This register-based cohort study aimed to examine the association between pre-TIA PA levels and the hazard of subsequent cerebrovascular events. Most participants engaged in light PA prior to the index TIA. Although moderate/vigorous PA was associated with lower hazards of ischaemic and total stroke in crude analysis, these associations were attenuated after adjustment for age and sex and remained similar with further adjustment. Overall, there was no clear evidence of an association between pre-TIA PA and subsequent cerebrovascular events after adjustment.

The observed attenuation after adjustment for age and sex likely reflects the substantial differences in baseline risk profiles across PA groups. Individuals with higher PA were generally younger and healthier, whereas sedentary participants were older and had a greater comorbidity burden. Another possible explanation may be reverse causality, as individuals with progressing disease may reduce PA prior to an event, making low PA a marker of elevated baseline risk rather than an independent risk factor. In our cohort, sedentary individuals were generally older with more comorbidities, raising the possibility that differential mortality could influence the observed associations. However, Fine-Gray analyses accounting for death as a competing event yielded estimates consistent with the primary Cox analyses, suggesting that competing mortality did not materially alter the adjusted associations.

Additional analyses supported the robustness of the findings with respect to age. There was no evidence of interaction between PA and age or of a nonlinear association between age and ischaemic stroke. Restricting the analysis to participants aged 60–79 years, where substantial age overlap was present across PA groups, also produced similar PA estimates. Thus, although age was an important confounder of the crude association, the attenuation observed after adjustment was unlikely to be explained solely by inadequate modelling of age or limited overlap between PA groups.

The lack of a significant association in our study is consistent with a previous study that found no association between pre-stroke PA and recurrence risk.^25^ In contrast, an international cohort study reported that regular PA prior to TIA or minor stroke was associated with a reduced 5-year risk of disabling stroke.^14^ This suggests that PA may be more strongly associated with the severity and functional outcome of subsequent events rather than with recurrence alone. Similarly, a narrative review examining the role of PA before and after stroke concluded that pre-stroke PA may be associated with lower stroke risk and severity and better functional outcomes, while post-stroke PA may reduce recurrence risk through improvements in established vascular risk factors, including blood pressure, glucose and lipid profiles.^15^ Taken together, these findings highlight the importance of considering the timing of PA assessment and the specific cerebrovascular outcome evaluated when comparing studies.

While ischaemic and total stroke outcomes demonstrated a gradual increase in cumulative incidence, stroke/TIA demonstrated a steep rise within the first days following the index event, irrespective of pre-TIA PA levels. This is consistent with the substantial early risk of recurrent TIA reported previously. In a prospective population-based study, 17% of TIA patients experienced a recurrent TIA within 7 days, of which 62% occurred within 24 hours and 80% within 48 hours.^26^ Previous research has shown that early stroke recurrence after TIA or minor stroke is associated with acute clinical and vascular risk factors, such as carotid stenosis or occlusion, systolic blood pressure, glucose levels and older age.^27^

Two secondary covariate associations were observed in the fully adjusted models. Female sex was associated with a higher hazard of the stroke/TIA composite, whereas no corresponding association was observed for ischaemic or total stroke, suggesting that this finding may primarily reflect recurrent TIA rather than stroke. However, as TIA was not analysed separately, this exploratory finding should be interpreted cautiously. Antiplatelet use was associated with a higher hazard of total stroke. However, this likely reflects, at least partly, confounding by indication, as antiplatelet users may have greater underlying vascular risk.^28^ Given the exploratory nature of the study and the number of secondary comparisons performed, both findings should be interpreted cautiously and should not be considered evidence of independent effects.

### Strengths and limitations

This study has several strengths. The use of linked regional and national registries enabled inclusion of a large, well-defined cohort of individuals with a registered TIA, with detailed information on sociodemographic factors, comorbidities, and medication use, enabling adjustment for several potential confounding factors and minimal loss to follow-up despite relocation within Sweden. The long follow-up period of up to 7 years enabled capture of both early and long-term recurrent events. The use of multiple outcome definitions strengthens the robustness and interpretability of the findings. Finally, the complementary analytical approaches allowed assessment of both relative associations and absolute risk over time.

Several limitations should be acknowledged as well. PA was self-reported and prone to social desirability bias, recall errors, and potential over/underestimation. Although SGPALS has demonstrated good validity for broadly categorising leisure-time PA, it is a relatively coarse self-reported ordinal measure, and misclassification between adjacent categories cannot be excluded. Such misclassification may have attenuated the observed associations. In addition, the relatively small size of the moderate/vigorous PA group may have reduced statistical power. Possible selection bias due to missing PA data should also be considered, as individuals with missing information may differ systematically from those included, potentially affecting the observed associations. A further limitation is potential reverse causality, where declining PA may reflect worsening health rather than contribute to recurrence. Residual confounding should also be considered, as registry-based data do not systematically capture all potentially relevant functional, behavioural, and vascular characteristics. While the CCI provides a measure of overall comorbidity burden, it cannot fully reflect differences in underlying health status across PA groups. Unmeasured or incompletely measured factors may therefore have influenced both PA levels and recurrence hazard. Potential administrative inconsistencies should be acknowledged, as some early recurrent events may have been misclassified. Lastly, as this is an observational study, causal inferences cannot be established, and the findings should therefore be interpreted with caution. The findings are likely generalisable to western populations with similar publicly funded healthcare systems.

## Conclusions

In this register-based cohort of individuals with TIA, higher pre-TIA PA was associated with substantially lower crude hazards of subsequent ischaemic and total stroke. These associations were attenuated after accounting for age and remained similar with further adjustment, suggesting that the lower observed risk among more active individuals was strongly related to differences in their baseline risk profile. Nevertheless, the estimates, particularly for moderate/vigorous PA, remained compatible with a potentially lower stroke hazard but were imprecise. Further studies incorporating more detailed and longitudinal measures of PA, functional status, and vascular risk are needed to clarify how PA patterns over time relate to cerebrovascular recurrence and outcomes.

## Supplementary material

10.1136/bmjopen-2026-124056 online supplemental file 1

## Data Availability

Data are available on reasonable request.
