# Supplementary material for: Physical activity prior to transient ischaemic attack and risk of subsequent cerebrovascular events: a Swedish register-based study
Source: BMJ Open. 2026 Sep 21;16(9):e124056. doi: 10.1136/bmjopen-2026-124056 (PMC13599882; doi:10.1136/bmjopen-2026-124056)
Supplement: Supplementary file 1 [file bmjopen-2026-124056.reviewer_comments.pdf]

PEER REVIEW HISTORY

BMJ Open publishes all reviews undertaken for accepted manuscripts. Reviewers are asked to complete a checklist review form and are provided with free text boxes to elaborate on their assessment. These free text comments are reproduced below.

ARTICLE DETAILS

Title (Provisional)

Physical Activity prior to Transient Ischemic Attack and Risk of Subsequent Cerebrovascular Events: A Swedish register based study

Authors

Beņislavska, Elīza Ance; Mavridis, Anastasios; Reinholdsson, Malin; Sunnerhagen, Katharina S

VERSION 1 - REVIEW

**Reviewer**	**1**
**Name**	**Sander, Dirk**
**Affiliation**	**Technische Universität München**
**Date**	**10-Jul-2026**
**COI**	**None**

This is a register-based longitudinal cohort study of 1,610 adults with a first registered TIA in Gothenburg, Sweden (2014–2019), linking a local stroke register (Väststroke) with Riksstroke, the National Patient Register, and Statistics Sweden. The authors examine the association between pre-TIA physical activity (PA), assessed with the four-level Saltin-Grimby Physical Activity Level Scale (SGPALS), and the hazard of subsequent ischemic stroke, total stroke, and a composite stroke/TIA outcome, using Cox proportional hazards models with progressive adjustment. A secondary, descriptive analysis uses Sankey diagrams to illustrate PA trajectories across recurrent events. The main finding is that a protective crude association between high PA and stroke recurrence is attenuated to non-significance after adjustment for age and sex, while descriptive PA-trajectory data suggest that sustained or increasing PA is associated with fewer/less severe recurrent events.

This manuscript addresses a clinically important and understudied question — the relationship between pre-TIA physical activity and recurrence risk. The cohort is large and well-characterized, and the use of linked national registries is a genuine strength. The core finding (attenuation of a crude protective association after adjustment) is a valuable reported result, and the descriptive PA-trajectory analysis is an interesting complementary angle. However, methodological clarity around missing data handling, competing risk of

death, confounder/mediator selection, and a few data-consistency issues in Table 1 should be addressed.

Major comments

1. The manuscript does not specify how long before the index TIA the SGPALS assessment refers to). Given the well-known risk of reverse causality that the authors themselves raise in the discussion, this needs to be stated explicitly in the Methods, and ideally the sensitivity of results to this ambiguity should be discussed further.

2. 315 eligible individuals were excluded for missing PA data, and additional cases were dropped in Models 3–4 (1,610 vs 1,568 analyzed). Only an age/sex comparison (Mann-Whitney U, chi-square) is presented for the PA-missing group; there is no comparison on clinical variables (comorbidity, atrial fibrillation, medication use) that might bias the adjusted estimates. A multiple imputation approach, or at minimum a missing-data comparison table, would strengthen confidence. Please also clarify whether Models 3–4 used list-wise deletion for the additional missing covariates (education, marital status, employment) and why marital/employment status, despite being collected and showing a clear gradient across PA groups in Table 1, were not included in the adjusted models.

3. The authors appropriately flag competing risk of death as a limitation but do not attempt any competing-risk analysis (e.g., Fine-Gray sub-distribution hazard, or cumulative incidence functions accounting for competing risk of death) despite this being standard in stroke recurrence literature with an elderly cohort where the sedentary group is both older and has higher comorbidity burden. Given this differential mortality risk is central to the authors' own explanation for the attenuation of the PA association, at least a sensitivity analysis using a competing-risks framework is warranted, or the Kaplan-Meier cumulative incidence curves in Figure 2 should be re-derived as cumulative incidence functions rather than 1−KM estimates.

4. It is not clear how the covariates in Model 3 (CCI, atrial fibrillation, education, country of birth) were chosen, A brief rationale for the adjustment sets used in each model would help readers judge whether residual/over-adjustment concerns apply.

5. The high-PA group is small (n=193, 12%), which the authors note reduces power; however, the manuscript reports point estimates and p-values across 3 outcomes × 4 models × multiple covariates without correction for multiple comparisons. Given the exploratory nature of the study this may be acceptable, but the authors should state explicitly that the analysis is exploratory/hypothesis-generating and that p-values should be interpreted with this in mind, particularly for secondary covariate associations highlighted in the discussion (e.g., anti-platelet use and total stroke, sex and stroke/TIA).

6. Female sex is associated with a significantly higher hazard of the composite stroke/TIA outcome (HR 1.30, Model 4) but not of ischemic or total stroke alone. This asymmetry is not discussed. Please comment on whether this may reflect a higher recurrent TIA (rather than

stroke) rate among women, and whether this has any implications for interpreting the composite outcome.

7. Please check the education-level breakdown for the sedentary group in Table 1 — the sedentary column reads "186 (32.4), 45 (42.7), 143 (24.9)" for lowest/secondary/highest education, but 186+45+143 = 374, not 606 (the total sedentary n), even though the listed percentages sum to 100%. This suggests either a transcription/formatting error or a genuine data error that should be corrected prior to publication.

8. The STROBE checklist submitted with the manuscript leaves item 12(e) ("sensitivity analyses") blank with no page number. Given the multiple limitations noted (missing data, competing risk of death, crude 4-level PA measure), at least one sensitivity analysis (e.g., multiple imputation for missing PA/covariates, or a competing-risks model) would substantially strengthen the paper's conclusions.

Minor comments

1. Section header "STRENGHTS AND LIMITATIONS" should read "STRENGTHS AND LIMITATIONS."

2. In the Results (page 16), "anti-platelet use was associated with higher hazard of total stroke (HR=1.59%, CI 95% 1.05-2.42)" — the "%" after 1.59 appears to be a typographical error and should be removed.

3. Please define the exact calendar window and definition used for "first registered TIA" more precisely relative to the one-year look-back period used to exclude prior stroke in the NPR.

4. The abstract's "Results" section could more explicitly state that the crude association did not persist after adjustment for age and sex alone (Model 2), not only after full adjustment, since this is the key take-home message and currently only stated for "adjusted analyses" generically.

5. Consider reporting median/IQR follow-up time and total person-years, which are commonly expected for register-based cohort papers (STROBE item 14c) but are not explicitly stated in the Results.

6. Figure 2 and Figure 3 legends should include brief explanatory text on how to read the Sankey diagram columns (e.g., what each node represents), since the description in the Methods, while present, is not repeated near the figure itself.

7. Please consider providing absolute risk differences (e.g., cumulative incidence at 1, 3, and 5 years by PA group) alongside hazard ratios, to aid clinical interpretability, as encouraged by STROBE item 16(c).

**Reviewer**	**2**
**Name**	**Kinoshita, Shoji**

Affiliation **The Jikei University School of Medicine, Department of Rehabilitation Medicine**

Date **14-Jul-2026**

COI **None**

This study addresses a clinically relevant question by examining the association between physical activity before transient ischemic attack and subsequent cerebrovascular events using linked regional and national registry data. The relatively large cohort and the availability of long-term follow-up are important strengths. However, I have substantial concerns regarding the formulation of the research question, the handling of confounding, the marked age imbalance across physical activity groups, the combination of early and late recurrent events, and particularly the interpretation of the physical activity transition analysis. These issues directly affect the validity of the main conclusions.

1. The research question and target estimand need to be clarified

The manuscript appears to combine three different research questions: whether pre-TIA physical activity causally reduces subsequent cerebrovascular events, whether pre-TIA physical activity is an independent prognostic factor, and whether changes in physical activity after TIA are associated with recurrence. These questions require different designs, covariate strategies, and interpretations, but the manuscript does not clearly distinguish among them or state a prespecified hypothesis.

The primary analyses show that the crude associations disappear after adjustment for age and sex. The manuscript then shifts emphasis toward the descriptive transition analysis and concludes that maintaining or increasing physical activity may be important for secondary prevention. This creates a disconnect between the primary results and the final clinical message.

The authors should clearly define whether the study aims to estimate a causal effect, assess an independent prognostic association, or evaluate predictive value. The hypothesis, covariate selection, statistical analysis, and conclusions should then be aligned with that objective.

2. The marked age imbalance across physical activity groups is a central concern

The mean age differed substantially across physical activity categories: 75.0 years in the sedentary group, 72.1 years in the light/moderate group, and 66.9 years in the high-activity group. The crude association between physical activity and recurrent stroke was no longer statistically significant after adjustment for age and sex. This suggests that the observed crude association may largely reflect the fact that younger and healthier participants were more active and also had a lower risk of recurrence.

Simply including age as a linear covariate may not adequately address this issue. The authors should present the full age distribution within each physical activity category and report the

number of participants and events by age group. They should also assess non-linearity in the association between age and outcome, examine interaction between age and physical activity, and provide age-stratified analyses. If high physical activity is uncommon among older participants, there may be insufficient common support across exposure groups, in which case conventional regression adjustment may not provide reliable estimates. A sensitivity analysis restricted to an age range in which all physical activity categories are adequately represented would be informative.

3. The rationale for covariate selection is unclear

The adjusted models include the Charlson Comorbidity Index, atrial fibrillation, education, country of birth, and several medication variables. In contrast, smoking is available in the dataset but is not included in the models, despite clear differences across physical activity groups and its likely association with recurrent vascular events.

More importantly, several clinically relevant confounders appear to be unavailable or unaccounted for, including pre-TIA functional status, mobility, frailty, cognitive impairment, severity and control of hypertension and diabetes, obesity, prior cardiovascular events, physical activity-limiting cardiac, pulmonary, neurological, or musculoskeletal disease, and the mechanism of the index TIA. Information on carotid stenosis, intracranial arterial disease, plaque characteristics, and acute secondary prevention would also be highly relevant.

The Charlson Comorbidity Index is a general measure of comorbidity burden and does not adequately capture the functional and vascular factors that may jointly influence physical activity and recurrent stroke risk.

The inclusion of atrial fibrillation together with anticoagulant use, and vascular disease-related treatment together with antiplatelet use, also requires justification. These variables represent a mixture of disease status, treatment, and treatment indication. A low variance inflation factor does not establish that the covariate set is clinically or causally appropriate. The authors should explain why education and country of birth were included, why smoking and individual vascular risk factors were omitted, and whether the adjustment set was prespecified on the basis of a clinical or causal framework.

4. Early and late recurrent events should not be treated as a single homogeneous outcome period without further justification

The Kaplan–Meier curve for the composite outcome of stroke or TIA shows a steep increase immediately after the index TIA. Very early recurrence is likely to be driven mainly by TIA mechanism, unstable vascular lesions, atrial fibrillation, acute antithrombotic treatment, carotid intervention, and possible duplication or misclassification of registry events. These determinants are clinically distinct from those influencing late recurrence.

At the same time, a single assessment of pre-TIA physical activity may not adequately represent exposure over a follow-up period of up to seven years, during which physical activity, frailty, vascular risk factors, and treatment are likely to change substantially.

The authors should justify the use of a single proportional hazard estimate across the entire follow-up period. Analyses separating very early, early, and late recurrence would be more clinically interpretable. A landmark analysis beginning at 90 days, or interval-specific analyses such as 0–7 days, 8–90 days, and beyond 90 days, should be considered. Fixed follow-up horizons such as 1, 3, or 5 years may also provide more stable and clinically meaningful estimates than relying on the extreme tail of the seven-year follow-up.

5. The physical activity transition analysis cannot support the conclusions drawn from it

The transition analysis does not represent longitudinal follow-up of physical activity in the full cohort. Repeat physical activity measurements were obtained only when participants were re-registered following another stroke or TIA. Participants without recurrent events therefore did not have comparable follow-up physical activity assessments.

In addition, the Sankey diagram includes only participants who had already experienced at least one recurrent event. The observation of physical activity is therefore dependent on the occurrence of the outcome itself. This design cannot be used to infer that maintaining or increasing physical activity is associated with fewer recurrent events, nor can it establish that longitudinal physical activity patterns provide information beyond baseline physical activity.

At most, the analysis describes changes in self-reported physical activity among patients who had already experienced recurrence. The secondary aim, the Sankey analysis, and the corresponding statements in the Abstract, Discussion, and Conclusion should therefore be removed or substantially restricted to a purely descriptive interpretation. The current data do not support conclusions regarding the preventive effect of maintaining or increasing physical activity.

6. The measurement and interpretation of SGPALS require further clarification

SGPALS is a coarse, self-reported ordinal measure of leisure-time physical activity. The Level 2 category primarily reflects light activity for at least four hours per week and should not be interpreted as equivalent to moderate-intensity physical activity as defined in current physical activity guidelines. The term “light/moderate physical activity” may therefore be misleading and should be reconsidered.

The manuscript should also clarify the reference period for physical activity recall, the timing of assessment after the TIA, whether the scale was self-administered or interviewer-administered, whether proxy responses were permitted, and how participants with cognitive impairment, language deficits, or acute neurological symptoms were handled. The practical distinction between sedentary and Level 2 activity should be explained.

Given the older TIA population and the subjective distinction between sedentary and light activity, exposure misclassification is likely. The authors should discuss more explicitly

whether SGPALS has adequate validity for distinguishing these categories in this population and how nondifferential misclassification may have attenuated the observed associations.

7. The clinical heterogeneity of the outcomes should be reconsidered

Ischemic stroke, total stroke, and the composite of stroke or TIA represent clinically and pathophysiologically different outcomes. Hemorrhagic stroke is not directly analogous to ischemic recurrence after TIA, and recurrent TIA is more susceptible to diagnostic uncertainty and registry duplication.

The rationale for classifying ICD-10 code I64 as ischemic stroke should be provided, ideally with reference to Swedish registry coding practice or validation data. The authors should consider defining ischemic stroke as the primary outcome and treating total stroke and stroke/TIA as secondary or exploratory outcomes. Sensitivity analyses excluding I64 would strengthen the interpretation, and the absolute number of hemorrhagic strokes should be reported.

The discussion of the association between antiplatelet use and total stroke should also be revised. Interpreting this finding as a possible hemorrhagic effect of antiplatelet treatment is not well supported without a separate analysis of hemorrhagic events and is more plausibly explained by confounding by indication.

**Reviewer**	**3**
**Name**	**Anderson, Craig**
**Affiliation**	**George Institute for Global Health**
**Date**	**26-Jul-2026**
**COI**	**None**

This manuscript uses regional and national record data linked to clinical information obtained through stroke unit admission of patients with transient ischemic attack (TIA) to a single university hospital in Gothenburg Sweden during 2014-2019 to determine associations between pre-morbid levels of activity and recurrent stroke and serious cardiovascular events. A total of 1610 TIA cases were included with no clear association with the outcomes in models adjusted for potential confounding variables.

The information is important for public health and clinical care.

The limitations of the analyses have been well articulated in a clearly presented and discussion report.

I only have one comment:

1) It is not clear of the relation of multiple stroke units to a single university hospital - please better define.

VERSION 1 - AUTHOR RESPONSE

Reviewer: 1

Dr. Dirk Sander, Technische Universität München

Comments to the Author:

This is a register-based longitudinal cohort study of 1,610 adults with a first registered TIA in Gothenburg, Sweden (2014–2019), linking a local stroke register (Väststroke) with Riksstroke, the National Patient Register, and Statistics Sweden. The authors examine the association between pre-TIA physical activity (PA), assessed with the four-level Saltin-Grimby Physical Activity Level Scale (SGPALS), and the hazard of subsequent ischemic stroke, total stroke, and a composite stroke/TIA outcome, using Cox proportional hazards models with progressive adjustment. A secondary, descriptive analysis uses Sankey diagrams to illustrate PA trajectories across recurrent events. The main finding is that a protective crude association between high PA and stroke recurrence is attenuated to non-significance after adjustment for age and sex, while descriptive PA-trajectory data suggest that sustained or increasing PA is associated with fewer/less severe recurrent events.

This manuscript addresses a clinically important and understudied question — the relationship between pre-TIA physical activity and recurrence risk. The cohort is large and well-characterized, and the use of linked national registries is a genuine strength. The core finding (attenuation of a crude protective association after adjustment) is a valuable reported result, and the descriptive PA-trajectory analysis is an interesting complementary angle. However, methodological clarity around missing data handling, competing risk of death, confounder/mediator selection, and a few data-consistency issues in Table 1 should be addressed.

Major comments

1. The manuscript does not specify how long before the index TIA the SGPALS assessment refers to). Given the well-known risk of reverse causality that the authors themselves raise in the discussion, this needs to be stated explicitly in the Methods, and ideally the sensitivity of results to this ambiguity should be discussed further.

Response: We thank the reviewer for highlighting the importance of the temporal reference of the PA assessment. We have clarified this substantially in the revised Methods. PA was assessed at the first encounter with a physiotherapist at the stroke unit, and participants were asked to estimate their average leisure-time PA before the event, generally referring to approximately the preceding year:

"PA was assessed in a standardized manner at the first encounter with a physiotherapist at the stroke unit. Participants were asked to estimate their average leisure-time PA before the event, generally referring to approximately the preceding year, with follow-up questions regarding intensity, duration, and type of activity. Relatives could assist in confirming the reported PA level when needed."

Because only one pre-TIA PA assessment was available, sensitivity analyses using alternative exposure periods were not possible. We nevertheless acknowledge the possibility of reverse causality, where reduced PA during the period preceding TIA may reflect declining underlying health rather than an independent risk factor, and this is discussed as a limitation.

2. 315 eligible individuals were excluded for missing PA data, and additional cases were dropped in Models 3–4 (1,610 vs 1,568 analyzed). Only an age/sex comparison (Mann-Whitney U, chi-square) is presented for the PA-missing group; there is no comparison on clinical variables (comorbidity, atrial fibrillation, medication use) that might bias the adjusted estimates. A multiple imputation approach, or at minimum a missing-data comparison table, would strengthen confidence. Please also clarify whether Models 3–4 used list-wise deletion for the additional missing covariates (education, marital status, employment) and why marital/employment status, despite being collected and showing a clear gradient across PA groups in Table 1, were not included in the adjusted models.

Response: We thank the reviewer for this suggestion. We expanded the missing-data analysis to compare participants with and without available PA data on demographic, socioeconomic, clinical, and treatment characteristics. The groups were broadly similar, although participants with missing PA were less frequently treated with antihypertensive medication (49% vs 56%, p=0.021) and lipid-lowering medication (18% vs 27%, p=0.001). These results are now presented in Supplementary Table 1, and the methods and results section have been modified accordingly:

"Participants with available and missing PA data were compared to assess potential selection associated with missing exposure information. Continuous variables were compared using the Mann-Whitney U test, while categorical demographic, socioeconomic, clinical, and treatment characteristics were compared using Pearson's chi-square test."

"Participants with and without available PA data were similar across demographic, socioeconomic, and clinical characteristics, including age, sex, comorbidity, atrial fibrillation, smoking, education, marital status, employment status, country of birth, antiplatelet use, and anticoagulant use (Supplementary Table 1). Participants with missing PA data were less frequently treated with antihypertensive medication (49% vs 56%, p = 0.021) and lipid-lowering medication (18% vs 27%, p = 0.001)."

We did not perform multiple imputation because PA was the primary exposure and was self-reported, with inherent potential for recall and social desirability bias. We therefore considered that missing PA values could not be reliably reconstructed from the available registry covariates without strong and unverifiable assumptions.

We have also clarified that Models 3-4 used complete-case analysis, resulting in 1,568 participants.

"All models were fitted separately for each outcome using complete-case analysis for the covariates included in each model. Consequently, participants with missing covariate information were excluded from Models 3 and 4, resulting in 1,568 participants in these models compared with 1,610 in Models 1 and 2."

Regarding marital and employment status, these variables were not included in the primary models as their relationship with physical activity levels and stroke outcomes is not clearly established. More specifically, in this older population, both marital and employment status are strongly influenced by age-related circumstances, including retirement and widowing, and may not specifically reflect broader socioeconomic status. Therefore, education level was retained as the principal socioeconomic factor. Nevertheless, in response to the reviewer's concern, an exploratory analysis was performed including both marital and employment status in the adjustment set. Neither variable was significantly associated with the risk of ischemic stroke, while the estimates for physical activity were virtually unchanged (light: HR 0.92, 95% CI 0.63–1.35; moderate/vigorous: HR 0.52, 95% CI 0.23-1.17). As their inclusion did not materially affect the estimates of interest, we did not add these variables to the primary models presented in the revised manuscript.

3. The authors appropriately flag competing risk of death as a limitation but do not attempt any competing-risk analysis (e.g., Fine-Gray sub-distribution hazard, or cumulative incidence functions accounting for competing risk of death) despite this being standard in stroke recurrence literature with an elderly cohort where the sedentary group is both older and has higher comorbidity burden. Given this differential mortality risk is central to the authors' own explanation for the attenuation of the PA association, at least a sensitivity analysis using a competing-risks framework is warranted, or the Kaplan-Meier cumulative incidence curves in Figure 2 should be re-derived as cumulative incidence functions rather than 1−KM estimates.

Response: We thank the reviewer for this important suggestion. We have now performed Fine-Gray competing-risk regression for all three outcomes, treating death as a competing event. The resulting subdistribution hazard estimates were broadly consistent in direction and magnitude with the primary Cox regression estimates and did not materially alter the conclusions. In addition, the original Kaplan-Meier curves in Figure 2 have been replaced with Aalen-Johansen cumulative incidence curves accounting for competing death, with 95% confidence intervals and numbers at risk. Absolute cumulative incidence estimates at prespecified time points are also provided in Table 2. The Methods, Results, Discussion, and corresponding limitation have been revised accordingly:

"Cumulative incidence of ischemic stroke, total stroke, and stroke or TIA was estimated across PA levels using the Aalen-Johansen estimator, with death treated as a competing event. Cumulative incidence functions are presented with 95% confidence intervals and numbers at risk. Absolute cumulative incidence estimates with 95% confidence intervals were additionally calculated at 7, 30, and 90 days and at 1, 3, and 5 years after the index TIA."

"To account for death as a competing event, Fine-Gray regression was additionally performed for each outcome to estimate subdistribution hazard ratios (sHRs) with 95% confidence intervals. The same four sequential adjustment models as in the Cox regression analyses were applied."

"Aalen-Johansen cumulative incidence curves accounting for death as a competing event are presented in Figure 2. For ischemic stroke, cumulative incidence during the first 90 days was similar across PA groups, reaching 1.5% (95% CI 0.7-2.7) in sedentary participants, 1.6% (95% CI 0.9-2.7) in those with light PA, and 1.6% (95% CI 0.4-4.2) in those with moderate/vigorous PA. During longer follow-up, the moderate/vigorous PA group showed greater separation from the

sedentary and light PA groups, although confidence intervals overlapped and estimates for the moderate/vigorous PA group were imprecise.

A similar pattern was observed for total stroke, with relatively low cumulative incidence early after the index TIA and greater separation of the moderate/vigorous PA group during longer follow-up. In contrast, the stroke/TIA composite showed a steep increase in cumulative incidence during the initial days following the index TIA across all PA groups, followed by a more gradual increase over time. Differences between PA groups were less pronounced for this composite outcome, with substantial overlap of the confidence intervals. Absolute cumulative incidence estimates at 7, 30, and 90 days and at 1, 3, and 5 years are presented in Table 2."

"Fine-Gray regression accounting for death as a competing event yielded results consistent with the Cox analyses (Supplementary Tables 2-4). For ischemic stroke, moderate/vigorous PA was associated with lower subdistribution hazard in the crude model (sHR 0.41, 95% CI 0.19-0.90). The association was attenuated after adjustment for age and sex (sHR 0.56, 95% CI 0.25-1.23) and remained similar in the fully adjusted model (sHR 0.57, 95% CI 0.25-1.28). Similar attenuation after adjustment was observed for total stroke, while no association was observed for the stroke/TIA composite after adjustment."

"In our cohort, sedentary individuals were generally older with more comorbidities, raising the possibility that differential mortality could influence the observed associations. However, Fine-Gray analyses accounting for death as a competing event yielded estimates consistent with the primary Cox analyses, suggesting that competing mortality did not materially alter the adjusted associations."

The limitation regarding competing risk of death was removed.

4. It is not clear how the covariates in Model 3 (CCI, atrial fibrillation, education, country of birth) were chosen, A brief rationale for the adjustment sets used in each model would help readers judge whether residual/over-adjustment concerns apply.

Response: We thank the reviewer for this comment. We agree that the rationale for the sequential adjustment sets was not sufficiently clear. We have therefore revised the Methods to explain the reasoning behind the inclusion of covariates in each model and to clarify the purpose of the progressive adjustment strategy.

"Given the exploratory observational design, sequential PH models were fitted to examine how the association between PA and subsequent events changed with progressive adjustment. Model 1 included PA only. Model 2 additionally included age and sex because of their established relationships with both PA and cerebrovascular risk. Model 3 further included CCI to capture overall comorbidity burden, atrial fibrillation as an established stroke risk factor, and education and country of birth as indicators of socioeconomic circumstances. Model 4 additionally included antihypertensive, lipid-lowering, antiplatelet, and anticoagulant medication use to account for differences in cardiovascular treatment and underlying vascular risk."

5. The high-PA group is small (n=193, 12%), which the authors note reduces power; however, the manuscript reports point estimates and p-values across 3 outcomes × 4 models × multiple covariates without correction for multiple comparisons. Given the exploratory nature of the

study this may be acceptable, but the authors should state explicitly that the analysis is exploratory/hypothesis-generating and that p-values should be interpreted with this in mind, particularly for secondary covariate associations highlighted in the discussion (e.g., anti-platelet use and total stroke, sex and stroke/TIA).

Response: We agree that the number of comparisons warrants caution when interpreting individual p-values. Given the exploratory and hypothesis-generating nature of the study, we did not apply a formal adjustment for multiple comparisons. In accordance with recent methodological discussions (Hooper R. (2025). To adjust, or not to adjust, for multiple comparisons. Journal of clinical epidemiology, 180, 111688. https://doi.org/10.1016/j.jclinepi.2025.111688) , we instead emphasize the context of the tested hypotheses, effect estimates, and 95% confidence intervals rather than statistical significance alone. We have clarified this explicitly in the Statistical Analysis section and have revised the interpretation of secondary covariate associations to avoid overemphasizing isolated statistically significant findings.

"All statistical tests were 2-tailed and performed at α 5%. Given the exploratory and hypothesis-generating nature of the study, no adjustment for multiple comparisons was applied. P-values were interpreted alongside effect estimates and 95% confidence intervals, and findings from secondary covariates should be interpreted cautiously."

"Two secondary covariate associations were observed in the fully adjusted models. Female sex was associated with a higher hazard of the stroke/TIA composite, whereas no corresponding association was observed for ischemic or total stroke, suggesting that this finding may primarily reflect recurrent TIA rather than stroke. However, as TIA was not analyzed separately, this exploratory finding should be interpreted cautiously. Antiplatelet use was associated with a higher hazard of total stroke. However, this likely reflects, at least partly, confounding by indication, as antiplatelet users may have greater underlying vascular risk. Given the exploratory nature of the study and the number of secondary comparisons performed, both findings should be interpreted cautiously and should not be considered evidence of independent effects."

6. Female sex is associated with a significantly higher hazard of the composite stroke/TIA outcome (HR 1.30, Model 4) but not of ischemic or total stroke alone. This asymmetry is not discussed. Please comment on whether this may reflect a higher recurrent TIA (rather than stroke) rate among women, and whether this has any implications for interpreting the composite outcome.

Response: We thank the reviewer for highlighting this finding. We have now discussed it together with the other secondary covariate findings. The association with female sex was observed for the stroke/TIA composite but not for ischemic or total stroke, suggesting that it may primarily reflect differences in recurrent TIA rather than stroke. However, because TIA was not analysed separately, this cannot be established from our data. We have therefore emphasized that this secondary finding, and the composite outcome more generally, should be interpreted cautiously given its heterogeneous composition and the exploratory nature of the analysis.

7. Please check the education-level breakdown for the sedentary group in Table 1 — the sedentary column reads "186 (32.4), 45 (42.7), 143 (24.9)" for lowest/secondary/highest

education, but 186+45+143 = 374, not 606 (the total sedentary n), even though the listed percentages sum to 100%. This suggests either a transcription/formatting error or a genuine data error that should be corrected prior to publication.

Response: We thank the reviewer for identifying this transcription error. The correct value for secondary education in the sedentary group is 245, rather than 45. The underlying data and analyses were correct, and Table 1 has been corrected accordingly.

8. The STROBE checklist submitted with the manuscript leaves item 12(e) ("sensitivity analyses") blank with no page number. Given the multiple limitations noted (missing data, competing risk of death, crude 4-level PA measure), at least one sensitivity analysis (e.g., multiple imputation for missing PA/covariates, or a competing-risks model) would substantially strengthen the paper's conclusions.

Response: We thank the reviewer for this suggestion. We have added several sensitivity analyses, including Fine-Gray competing-risk regression, assessment of age overlap, PA × age interaction and age non-linearity, and analyses restricted to participants aged 60-79 years. We also expanded the comparison of participants with and without available PA data. These analyses did not materially alter the main findings. The STROBE checklist, including item 12(e), has been updated accordingly.

Minor comments

1. Section header "STRENGHTS AND LIMITATIONS" should read "STRENGTHS AND LIMITATIONS."
   Response: We thank the reviewer for noticing this. The error has been corrected.

2. In the Results (page 16), "anti-platelet use was associated with higher hazard of total stroke (HR=1.59%, CI 95% 1.05-2.42)" — the "%" after 1.59 appears to be a typographical error and should be removed.
   Response: Thank you for noticing this, the typographical error has been corrected.

3. Please define the exact calendar window and definition used for "first registered TIA" more precisely relative to the one-year look-back period used to exclude prior stroke in the NPR.
   Response: We thank the reviewer for highlighting this. We have clarified the definition of the index TIA in the Methods. All TIA registrations in Väststroke during the study period were considered, with the first registration during this period defined as the index TIA. The one-year NPR look-back period for previous stroke was applied relative to this index event.
   "The study population was identified through the local stroke and TIA register Väststroke between November 1, 2014, and December 29, 2019. Adult patients (≥18 years) with a TIA (ICD-10 G45.9) registered in Väststroke during the study period were included, with the first registration during this period defined as the index event. Individuals were excluded if they had a previous diagnosis of ischemic stroke (ICD-10 I63), unspecified stroke (ICD-10 I64) or hemorrhagic stroke (ICD-10 I61) recorded in Väststroke prior to the index date during the study period or in the NPR within one year prior to the index date." Methods pg. 5

4. The abstract's "Results" section could more explicitly state that the crude association did not persist after adjustment for age and sex alone (Model 2), not only after full adjustment, since this is the key take-home message and currently only stated for "adjusted analyses" generically.
   Response: We thank the reviewer for this suggestion. We agree that attenuation following adjustment for age and sex represents an important finding. We have revised the Abstract accordingly:
   "These associations were attenuated after adjustment for age and sex and were no longer statistically significant (HR 0.48, 95% CI 0.22 to 1.08 for ischemic stroke; HR 0.53, 95% CI 0.26 to 1.09 for total stroke). Further adjustment for clinical, socioeconomic, and treatment-related factors did not materially alter the estimates."

5. Consider reporting median/IQR follow-up time and total person-years, which are commonly expected for register-based cohort papers (STROBE item 14c) but are not explicitly stated in the Results.
   Response: We thank the reviewer for this suggestion. We have added the median and interquartile range of follow-up and total person-years for each outcome to the Results section.
   "Median follow-up was 3.89 years (IQR 2.63-5.39) for ischemic stroke, corresponding to 6,329 person-years. Corresponding follow-up was 3.88 years (IQR 2.62-5.37; 6,316 person-years) for total stroke and 3.34 years (IQR 2.08-5.11; 5,462 person-years) for stroke/TIA."

6. Figure 2 and Figure 3 legends should include brief explanatory text on how to read the Sankey diagram columns (e.g., what each node represents), since the description in the Methods, while present, is not repeated near the figure itself.

Response: We thank the reviewer for this suggestion. The Sankey diagram has now been moved to the Supplementary Material, and its legend has been expanded to explain the sequential PA and event columns and the connecting flows. The Figure 2 legend has also been updated to reflect the revised Aalen-Johansen cumulative incidence curves.

7. Please consider providing absolute risk differences (e.g., cumulative incidence at 1, 3, and 5 years by PA group) alongside hazard ratios, to aid clinical interpretability, as encouraged by STROBE item 16(c).

Response: We thank the reviewer for this suggestion. To improve clinical interpretability, we have added absolute cumulative incidence estimates with 95% confidence intervals for each PA group at 7, 30, and 90 days and at 1, 3, and 5 years. These estimates were derived using the Aalen-Johansen estimator with death treated as a competing event and are presented in Table 2.

Reviewer: 2

Dr. Shoji Kinoshita, The Jikei University School of Medicine

Comments to the Author:

This study addresses a clinically relevant question by examining the association between physical activity before transient ischemic attack and subsequent cerebrovascular events using linked regional and national registry data. The relatively large cohort and the availability of long-term follow-up are important strengths. However, I have substantial concerns regarding the

formulation of the research question, the handling of confounding, the marked age imbalance across physical activity groups, the combination of early and late recurrent events, and particularly the interpretation of the physical activity transition analysis. These issues directly affect the validity of the main conclusions.

1. The research question and target estimand need to be clarified

The manuscript appears to combine three different research questions: whether pre-TIA physical activity causally reduces subsequent cerebrovascular events, whether pre-TIA physical activity is an independent prognostic factor, and whether changes in physical activity after TIA are associated with recurrence. These questions require different designs, covariate strategies, and interpretations, but the manuscript does not clearly distinguish among them or state a prespecified hypothesis.

The primary analyses show that the crude associations disappear after adjustment for age and sex. The manuscript then shifts emphasis toward the descriptive transition analysis and concludes that maintaining or increasing physical activity may be important for secondary prevention. This creates a disconnect between the primary results and the final clinical message.

The authors should clearly define whether the study aims to estimate a causal effect, assess an independent prognostic association, or evaluate predictive value. The hypothesis, covariate selection, statistical analysis, and conclusions should then be aligned with that objective.

Response:

We thank the reviewer for this important comment and agree that the original manuscript did not sufficiently distinguish the primary research question from the exploratory analysis of PA changes. We have therefore substantially reframed and revised the manuscript to clarify that the primary objective is to examine the association between pre-TIA PA and subsequent cerebrovascular events, rather than to estimate a causal effect or predictive performance. Ischemic stroke is now explicitly defined as the primary outcome, with total stroke and stroke/TIA as secondary outcomes. The Methods, Results, Discussion, and Conclusions have been revised accordingly to align with this objective and to emphasize the attenuation of the crude associations after adjustment for age and sex. The PA-transition analysis has been reframed as exploratory and descriptive, moved to the Supplementary Material, and no longer informs the main clinical conclusions. Some key changes are highlighted below, but given the overarching nature of this comment, the manuscript has been revised throughout as appropriate to ensure consistency with the clarified research objective.

"In addition, evidence regarding pre-TIA PA levels and their association with subsequent cerebrovascular events remains limited.

The primary aim of this study was to examine the association between pre-TIA PA levels and subsequent cerebrovascular events following TIA, with ischemic stroke as the primary outcome and total stroke and stroke/TIA as secondary outcomes."

"*The primary outcome was ischemic stroke (ICD-10 I63 and I64). Secondary outcomes were total stroke (I61, I63, and I64), and a composite outcome of stroke or TIA (I61, I63, I64, and G45.9)."*

"Given the exploratory observational design, sequential PH models were fitted to examine how the association between PA and subsequent events changed with progressive adjustment."

"*In this register-based cohort of individuals with TIA, higher pre-TIA PA was associated with substantially lower crude hazards of subsequent ischemic and total stroke. These associations were attenuated after accounting for age and remained similar with further adjustment, suggesting that the lower observed risk among more active individuals was strongly related to differences in their baseline risk profile."*

2. The marked age imbalance across physical activity groups is a central concern

The mean age differed substantially across physical activity categories: 75.0 years in the sedentary group, 72.1 years in the light group, and 66.9 years in the moderate/vigorous activity group. The crude association between physical activity and recurrent stroke was no longer statistically significant after adjustment for age and sex. This suggests that the observed crude association may largely reflect the fact that younger and healthier participants were more active and also had a lower risk of recurrence.

Simply including age as a linear covariate may not adequately address this issue. The authors should present the full age distribution within each physical activity category and report the number of participants and events by age group. They should also assess non-linearity in the association between age and outcome, examine interaction between age and physical activity, and provide age-stratified analyses. If high physical activity is uncommon among older participants, there may be insufficient common support across exposure groups, in which case conventional regression adjustment may not provide reliable estimates. A sensitivity analysis restricted to an age range in which all physical activity categories are adequately represented would be informative.

Response: We thank the reviewer for highlighting the substantial age differences across PA groups. We agree that this required more detailed assessment beyond conventional linear adjustment. We therefore performed several additional age-related sensitivity analyses. Age distributions across PA groups were examined using density plots, where significant overlap was observed among the three groups. Additionally, we assessed both PA × age interaction and non-linearity of age in the age- and sex-adjusted and fully adjusted models. There was no evidence of interaction (P for interaction=0.689 and 0.670, respectively) or non-linearity (P for non-linearity=0.536 and 0.368, respectively). We also examined common support across PA groups. Because overlap was substantially greater between ages 60-79 years, we repeated the Cox analyses restricted to this age range. The PA estimates remained similar to those in the full

cohort: in the age- and sex-adjusted model, HR 0.81 (95% CI 0.47-1.41) for light PA and HR 0.52 (95% CI 0.19-1.38) for moderate/vigorous PA; corresponding fully adjusted estimates were HR 0.80 (95% CI 0.45-1.42) and HR 0.57 (95% CI 0.21-1.56). We did not present separate age-stratified regression models because event counts were very small within individual age strata, resulting in insufficient precision for meaningful estimation. These additional sensitivity analyses have been added in the methods and results sections, and they are moreover discussed in the discussion section.

3. The rationale for covariate selection is unclear

The adjusted models include the Charlson Comorbidity Index, atrial fibrillation, education, country of birth, and several medication variables. In contrast, smoking is available in the dataset but is not included in the models, despite clear differences across physical activity groups and its likely association with recurrent vascular events.

More importantly, several clinically relevant confounders appear to be unavailable or unaccounted for, including pre-TIA functional status, mobility, frailty, cognitive impairment, severity and control of hypertension and diabetes, obesity, prior cardiovascular events, physical activity-limiting cardiac, pulmonary, neurological, or musculoskeletal disease, and the mechanism of the index TIA. Information on carotid stenosis, intracranial arterial disease, plaque characteristics, and acute secondary prevention would also be highly relevant.

The Charlson Comorbidity Index is a general measure of comorbidity burden and does not adequately capture the functional and vascular factors that may jointly influence physical activity and recurrent stroke risk.

The inclusion of atrial fibrillation together with anticoagulant use, and vascular disease-related treatment together with antiplatelet use, also requires justification. These variables represent a mixture of disease status, treatment, and treatment indication. A low variance inflation factor does not establish that the covariate set is clinically or causally appropriate. The authors should explain why education and country of birth were included, why smoking and individual vascular risk factors were omitted, and whether the adjustment set was prespecified on the basis of a clinical or causal framework.

Response: We thank the reviewer for this important comment and agree that the available covariates cannot constitute a comprehensive causal adjustment set for the relationship between PA and recurrent stroke. PA is a particularly complex exposure in this regard, as it is closely intertwined with age, comorbidity, functional status, frailty, socioeconomic circumstances, health behaviours, and treatment, which may have different roles as confounders, mediators, or markers of underlying health. This makes specification of an unambiguous causal framework challenging. As clarified in the revised manuscript, this was an exploratory observational study, and the sequential models were intended to examine how the

PA association changed with progressive adjustment rather than to estimate a formally specified causal effect. We have revised the Methods to explain the rationale for the adjustment sets:

"Given the exploratory observational design, sequential PH models were fitted to examine how the association between PA and subsequent events changed with progressive adjustment. Model 1 included PA only. Model 2 additionally included age and sex because of their established relationships with both PA and cerebrovascular risk. Model 3 further included CCI to capture overall comorbidity burden, atrial fibrillation as an established stroke risk factor, and education and country of birth as indicators of socioeconomic circumstances. Model 4 additionally included antihypertensive, lipid-lowering, antiplatelet, and anticoagulant medication use to account for differences in cardiovascular treatment and underlying vascular risk."

Smoking was not included in the primary models because of its relatively high proportion of missing data and its high susceptibility to social desirability bias. In response to the reviewer, we repeated the fully adjusted ischemic stroke model with additional adjustment for smoking. The PA estimates remained similar: HR 1.07 (95% CI 0.71-1.60) for light PA and HR 0.57 (95% CI 0.25-1.31) for moderate/vigorous PA, compared with HR 0.93 (95% CI 0.63-1.36) and HR 0.52 (95% CI 0.23-1.16), respectively, in the primary model.

We also agree that potentially important functional and vascular characteristics were unavailable, reflecting the inherent limitations of registry-based data. Although the CCI captures overall comorbidity burden, residual confounding by factors not captured in the registers cannot be excluded, and this has been emphasized in the revised limitations:

"Residual confounding should also be considered, as registry-based data do not systematically capture all potentially relevant functional, behavioral, and vascular characteristics. While the CCI provides a measure of overall comorbidity burden, it cannot fully reflect differences in underlying health status across PA groups. Unmeasured or incompletely measured factors may therefore have influenced both PA levels and recurrence hazard."

Atrial fibrillation was included as an established stroke risk factor, while medication variables were included to account for differences in cardiovascular treatment and underlying vascular risk. We acknowledge that medication use also reflects treatment indication and disease severity and therefore do not interpret these coefficients causally. Education and country of birth were included to account for socioeconomic and sociodemographic differences that may influence both PA patterns and cerebrovascular risk.

4. Early and late recurrent events should not be treated as a single homogeneous outcome period without further justification

The Kaplan–Meier curve for the composite outcome of stroke or TIA shows a steep increase immediately after the index TIA. Very early recurrence is likely to be driven mainly by TIA mechanism, unstable vascular lesions, atrial fibrillation, acute antithrombotic treatment, carotid intervention, and possible duplication or misclassification of registry events. These determinants are clinically distinct from those influencing late recurrence.

At the same time, a single assessment of pre-TIA physical activity may not adequately represent exposure over a follow-up period of up to seven years, during which physical activity, frailty, vascular risk factors, and treatment are likely to change substantially.

The authors should justify the use of a single proportional hazard estimate across the entire follow-up period. Analyses separating very early, early, and late recurrence would be more clinically interpretable. A landmark analysis beginning at 90 days, or interval-specific analyses such as 0–7 days, 8–90 days, and beyond 90 days, should be considered. Fixed follow-up horizons such as 1, 3, or 5 years may also provide more stable and clinically meaningful estimates than relying on the extreme tail of the seven-year follow-up.

Response: We thank the reviewer for this important comment. We agree that early and late recurrence may reflect different underlying mechanisms and that a single baseline PA assessment may become less representative over prolonged follow-up. We explored interval-specific Cox analyses but the small number of ischemic stroke events within the early follow-up periods resulted in insufficiently precise estimates, and we therefore did not consider these analyses reliable for presentation.

Instead, we added Aalen-Johansen absolute cumulative incidence estimates at clinically relevant fixed horizons of 7, 30, and 90 days and 1, 3, and 5 years, accounting for death as a competing event. This provides clinically interpretable estimates of absolute risk across early, intermediate, and longer-term follow-up without relying on unstable interval-specific regression estimates, while also allowing the temporal pattern of recurrence across PA groups to be assessed. The methods, results, and discussion section have been revised accordingly.

5. The physical activity transition analysis cannot support the conclusions drawn from it

The transition analysis does not represent longitudinal follow-up of physical activity in the full cohort. Repeat physical activity measurements were obtained only when participants were re-registered following another stroke or TIA. Participants without recurrent events therefore did not have comparable follow-up physical activity assessments.

In addition, the Sankey diagram includes only participants who had already experienced at least one recurrent event. The observation of physical activity is therefore dependent on the occurrence of the outcome itself. This design cannot be used to infer that maintaining or increasing physical activity is associated with fewer recurrent events, nor can it establish that longitudinal physical activity patterns provide information beyond baseline physical activity.

At most, the analysis describes changes in self-reported physical activity among patients who had already experienced recurrence. The secondary aim, the Sankey analysis, and the corresponding statements in the Abstract, Discussion, and Conclusion should therefore be removed or substantially restricted to a purely descriptive interpretation. The current data do not support conclusions regarding the preventive effect of maintaining or increasing physical activity.

Response: We agree with the reviewer that repeat PA measurements were conditional on subsequent Väststroke registrations and therefore do not constitute longitudinal PA follow-up of the full cohort. Consequently, this analysis cannot be used to assess whether maintaining or increasing PA is associated with recurrence risk.

We have substantially revised the manuscript accordingly. The PA-transition analysis is no longer presented as a secondary study aim and has been moved from the main analysis to the Supplementary Material, where it is explicitly described as an exploratory descriptive analysis restricted to participants with recurrent Väststroke registrations. Statements suggesting that maintaining or increasing PA was associated with fewer recurrent events or that longitudinal PA patterns provide information beyond baseline PA have been removed from the Abstract, Discussion, and Conclusions. The analysis is now interpreted solely as a description of observed PA transitions among participants with recurrent registrations.

6. The measurement and interpretation of SGPALS require further clarification

SGPALS is a coarse, self-reported ordinal measure of leisure-time physical activity. The Level 2 category primarily reflects light activity for at least four hours per week and should not be interpreted as equivalent to moderate-intensity physical activity as defined in current physical activity guidelines. The term “light/moderate physical activity” may therefore be misleading and should be reconsidered.

The manuscript should also clarify the reference period for physical activity recall, the timing of assessment after the TIA, whether the scale was self-administered or interviewer-administered, whether proxy responses were permitted, and how participants with cognitive impairment, language deficits, or acute neurological symptoms were handled. The practical distinction between sedentary and Level 2 activity should be explained.

Given the older TIA population and the subjective distinction between sedentary and light activity, exposure misclassification is likely. The authors should discuss more explicitly whether SGPALS has adequate validity for distinguishing these categories in this population and how nondifferential misclassification may have attenuated the observed associations.

Response: We thank the reviewer for this important comment. We have substantially expanded the description of the SGPALS assessment in the Methods. We now clarify that PA was assessed at the first encounter with a physiotherapist, generally referring to approximately the preceding year, with follow-up questions regarding activity intensity, duration, and type, and that relatives could assist in confirming the reported PA level when needed. We have also provided the full definitions and practical examples for each SGPALS level.

We agree that our previous terminology for Level 2 was potentially misleading. In accordance with the SGPALS definitions, Level 2 is now referred to as light PA, while the combined Levels 3-4 are referred to as moderate/vigorous PA throughout the manuscript. The Methods have also been expanded to describe the established concurrent and predictive validity of SGPALS.

Finally, we have expanded the limitations to acknowledge that, despite its demonstrated validity for broad categorization of leisure-time PA, SGPALS remains a relatively coarse self-reported ordinal measure. Misclassification between adjacent categories, particularly sedentary and light PA, cannot be excluded and may have attenuated the observed associations.

7. The clinical heterogeneity of the outcomes should be reconsidered

Ischemic stroke, total stroke, and the composite of stroke or TIA represent clinically and pathophysiologically different outcomes. Hemorrhagic stroke is not directly analogous to ischemic recurrence after TIA, and recurrent TIA is more susceptible to diagnostic uncertainty and registry duplication.

The rationale for classifying ICD-10 code I64 as ischemic stroke should be provided, ideally with reference to Swedish registry coding practice or validation data. The authors should consider defining ischemic stroke as the primary outcome and treating total stroke and stroke/TIA as secondary or exploratory outcomes. Sensitivity analyses excluding I64 would strengthen the interpretation, and the absolute number of hemorrhagic strokes should be reported.

The discussion of the association between antiplatelet use and total stroke should also be revised. Interpreting this finding as a possible hemorrhagic effect of antiplatelet treatment is not well supported without a separate analysis of hemorrhagic events and is more plausibly explained by confounding by indication.

Response: We thank the reviewer for this important comment and agree that the outcomes differ clinically and should not be given equivalent emphasis. We have therefore reframed ischemic stroke as the primary outcome, while total stroke and the stroke/TIA composite are now explicitly considered secondary outcomes throughout the manuscript.

We have also clarified the rationale for including I64 within the ischemic stroke definition. In contemporary Swedish stroke care, brain imaging is performed routinely; in the 2019 Riksstroke report, CT was performed in 98% of patients with ischemic stroke. Consequently, it is unlikely that a substantial number of intracerebral hemorrhages would remain classified as unspecified stroke. I64 is also uncommon, representing approximately 1% of all stroke registrations in Riksstroke. This rationale and the supporting Riksstroke reference have now been added to the Methods.

The absolute number of hemorrhagic events has also been reported in the study population subsection of the results:

"During follow-up, 124 participants experienced ischemic stroke, 137 experienced total stroke (including 13 hemorrhagic strokes), and 379 experienced stroke or TIA."

We also agree that our original interpretation of the association between antiplatelet use and total stroke placed undue emphasis on a possible hemorrhagic mechanism. Given the small number of hemorrhagic events and the observational design, this interpretation was not sufficiently supported. The Discussion has therefore been revised to emphasize confounding by indication and underlying vascular risk as more plausible explanations, and the antiplatelet association is now explicitly treated as an exploratory secondary finding:

"Antiplatelet use was associated with a higher hazard of total stroke. However, this likely reflects, at least partly, confounding by indication, as antiplatelet users may have greater underlying vascular risk. Given the exploratory nature of the study and the number of secondary comparisons performed, both findings should be interpreted cautiously and should not be considered evidence of independent effects."

Reviewer: 3

Prof. Craig Anderson, George Institute for Global Health

Comments to the Author:

This manuscript uses regional and national record data linked to clinical information obtained through stroke unit admission of patients with transient ischemic attack (TIA) to a single university hospital in Gothenburg Sweden during 2014-2019 to determine associations between pre-morbid levels of activity and recurrent stroke and serious cardiovascular events. A total of 1610 TIA cases were included with no clear association with the outcomes in models adjusted for potential confounding variables.

The information is important for public health and clinical care.

The limitations of the analyses have been well articulated in a clearly presented and discussion report.

I only have one comment:

1) It is not clear of the relation of multiple stroke units to a single university hospital - please better define.
   Response: We thank the reviewer for identifying this ambiguity. Sahlgrenska University Hospital represents a single university hospital organization comprising multiple hospital sites and stroke units in the Gothenburg area. We have clarified this organizational relationship in the Methods:
   "Väststroke includes all individuals with stroke or TIA treated at the stroke units at Sahlgrenska University Hospital, a university hospital organization comprising three hospital sites in the Gothenburg area."

VERSION 2 - REVIEW

**Reviewer**	**1**
**Name**	**Sander, Dirk**
**Affiliation**	**Technische Universität München**
**Date**	**27-Aug-2026**
**COI**	

The authors responded to my suggestions and comments sufficiently.

Reviewer 2

Name Kinoshita, Shoji

Affiliation The Jikei University School of Medicine, Department of Rehabilitation Medicine

Date 27-Aug-2026

COI

Thank you for the careful and substantial revision of the manuscript. The authors have responded constructively to my previous comments and have substantially improved the clarity and interpretation of the study.

In particular, the research objective is now appropriately framed as an observational association rather than a causal effect, with ischemic stroke clearly defined as the primary outcome. The additional analyses addressing the age imbalance, including assessment of age distribution, interaction and non-linearity, and the restricted-age sensitivity analysis, are helpful. The rationale for covariate adjustment and the limitations related to residual confounding are also more clearly presented. The additional competing-risk and fixed-time cumulative incidence analyses improve the interpretation of the follow-up data. Importantly, the PA transition analysis has been appropriately repositioned as exploratory and descriptive, and the previous clinical interpretation based on these transitions has been removed. The description and interpretation of the SGPALS categories and the definition and hierarchy of cerebrovascular outcomes have also been clarified.

Overall, I believe that my previous concerns have been adequately addressed, and I have no further comments.

Reviewer 3

Name Anderson, Craig

Affiliation George Institute for Global Health

Date 31-Aug-2026

COI

---

the authors have well addressed reviewer comments
