# Supplementary material for: Physical activity prior to transient ischaemic attack and risk of subsequent cerebrovascular events: a Swedish register-based study
Source: BMJ Open. 2026 Sep 21;16(9):e124056. doi: 10.1136/bmjopen-2026-124056 (PMC13599882; doi:10.1136/bmjopen-2026-124056)
Supplement: Supplementary file 2 [file bmjopen-16-9-s001.docx]

**ONLINE SUPPLEMENT**

**Physical Activity prior to Transient Ischemic Attack and Risk of Subsequent Cerebrovascular Events: A Swedish register‑based study**

*Elīza Ance Beņislavska^1,2^; *Anastasios Mavridis^1^, MD; Malin Reinholdsson^1,3^, PhD; Katharina S Sunnerhagen^1,3^, MD, PhD

**Affiliations**

^1^Department of Clinical Neuroscience, Institute of Neuroscience and Physiology, Sahlgrenska Academy, University of Gothenburg, Sweden

^2^Riga Stradins University, Riga, Latvia

^3^The Sahlgrenska University Hospital, Gothenburg, Sweden

*The authors contributed equally to this work and share first authorship

**Corresponding author**: Anastasios Mavridis, Institute of Neuroscience and Physiology, Rehabilitation Medicine, University of Gothenburg, Sweden, Vita stråket 12, fl. 4, 41345 Gothenburg, Sweden. +46735861950, [anastasios.mavridis@gu.se](mailto:anastasios.mavridis@gu.se)

**Supplementary Table 1.** Comparison of demographic, socioeconomic, clinical, and treatment characteristics between participants with available and missing pre-TIA physical activity data.

| **Characteristic** | **Overall**^1^ | **PA available**^1^ | **PA missing**^1^ | **p-value**^2^ |
| --- | --- | --- | --- | --- |
| **Age, years** | 73 (65, 81) | 73 (65, 81) | 72 (62, 81) | 0.058 |
| **Sex** |  |  |  | 0.727 |
| Male | 933 (48%) | 777 (48%) | 156 (50%) |  |
| Female | 992 (52%) | 833 (52%) | 159 (50%) |  |
| **Charlson Comorbidity Index** | 0.18 (0.65) | 0.17 (0.64) | 0.23 (0.69) | 0.159 |
| **Atrial fibrillation** | 355 (18%) | 290 (18%) | 65 (21%) | 0.309 |
| **Smoking** | 165 (9.5%) | 131 (9.0%) | 34 (12%) | 0.103 |
| Missing | 194 | 154 | 40 |  |
| **Education level** |  |  |  | 0.204 |
| Lowest | 504 (27%) | 427 (27%) | 77 (25%) |  |
| Secondary | 817 (44%) | 693 (44%) | 124 (41%) |  |
| Highest | 550 (29%) | 448 (29%) | 102 (34%) |  |
| Missing | 54 | 42 | 12 |  |
| **Marital status** |  |  |  | 0.481 |
| Partnered | 966 (51%) | 816 (52%) | 150 (49%) |  |
| No partner | 921 (49%) | 766 (48%) | 155 (51%) |  |
| Missing | 38 | 28 | 10 |  |
| **Employment status** |  |  |  | 0.246 |
| Employed | 485 (26%) | 398 (25%) | 87 (29%) |  |
| Unemployed | 1,402 (74%) | 1,184 (75%) | 218 (71%) |  |
| Missing | 38 | 28 | 10 |  |
| **Country of birth** |  |  |  | 0.236 |
| Sweden | 1,592 (83%) | 1,341 (83%) | 251 (80%) |  |
| EU without Sweden | 240 (12%) | 196 (12%) | 44 (14%) |  |
| Rest of world | 93 (4.8%) | 73 (4.5%) | 20 (6.3%) |  |
| **Antihypertensive medication** | 1,064 (55%) | 909 (56%) | 155 (49%) | 0.021 |
| **Lipid-lowering medication** | 483 (25%) | 427 (27%) | 56 (18%) | 0.001 |
| **Antiplatelet medication** | 396 (21%) | 335 (21%) | 61 (19%) | 0.615 |
| **Anticoagulant medication** | 229 (12%) | 187 (12%) | 42 (13%) | 0.443 |
| ^1^Median (Q1, Q3); n (%); Mean (SD); ^2^Mann-Whitney U test; Pearson's Chi-squared test; PA: Physical Activity | | | | |

**Supplementary Table 2.** Fine–Gray competing-risk regression models with varying adjustments for ischemic stroke, with death treated as a competing event.

|  | Model 1 | | Model 2 | | Model 3 | | Model 4 | |
| --- | --- | --- | --- | --- | --- | --- | --- | --- |
|  | sHR (95% CI) | p-value | sHR (95% CI) | p-value | sHR (95% CI) | p-value | sHR (95% CI) | p-value |
| Physical activity: Light | 0.90 (0.62 - 1.29) | 0.556 | 1.02 (0.70 - 1.48) | 0.921 | 1.01 (0.68 - 1.48) | 0.979 | 1.02 (0.69 - 1.50) | 0.925 |
| Physical activity: Moderate/Vigorous | 0.41 (0.19 - 0.90) | 0.026 | 0.56 (0.25 - 1.23) | 0.147 | 0.54 (0.24 - 1.22) | 0.137 | 0.57 (0.25 - 1.28) | 0.172 |
| Age | NA | NA | 1.04 (1.02 - 1.06) | <0.001 | 1.04 (1.02 - 1.06) | <0.001 | 1.03 (1.01 - 1.05) | <0.001 |
| Sex: Female | NA | NA | 0.97 (0.68 - 1.39) | 0.887 | 0.97 (0.67 - 1.41) | 0.879 | 1.00 (0.68 - 1.45) | 0.983 |
| CCI | NA | NA | NA | NA | 0.87 (0.64 - 1.17) | 0.355 | 0.84 (0.60 - 1.16) | 0.285 |
| Atrial fibrillation | NA | NA | NA | NA | 0.99 (0.62 - 1.56) | 0.949 | 0.86 (0.49 - 1.53) | 0.615 |
| Education: Secondary | NA | NA | NA | NA | 1.20 (0.77 - 1.85) | 0.421 | 1.20 (0.77 - 1.86) | 0.418 |
| Education: Higher | NA | NA | NA | NA | 1.06 (0.64 - 1.75) | 0.829 | 1.05 (0.63 - 1.74) | 0.848 |
| Country of birth: EU without Sweden | NA | NA | NA | NA | 0.93 (0.54 - 1.61) | 0.801 | 0.93 (0.54 - 1.61) | 0.804 |
| Country of birth: Rest of world | NA | NA | NA | NA | 0.86 (0.27 - 2.75) | 0.797 | 0.80 (0.25 - 2.56) | 0.708 |
| Antihypertensive medication | NA | NA | NA | NA | NA | NA | 1.47 (0.95 - 2.27) | 0.083 |
| Lipid-lowering medication | NA | NA | NA | NA | NA | NA | 0.81 (0.51 - 1.29) | 0.382 |
| Antiplatelet medication | NA | NA | NA | NA | NA | NA | 1.53 (0.95 - 2.44) | 0.078 |
| Anticoagulant medication | NA | NA | NA | NA | NA | NA | 1.23 (0.63 - 2.39) | 0.550 |
| **Total cases** | **1610** | | **1610** | | **1568** | | **1568** | |
| **Events** | **124** | | **124** | | **119** | | **119** | |
| **Competing events (death)** | **253** | | **253** | | **224** | | **224** | |
| sHR = subdistribution hazard ratio; CI = confidence interval; CCI = Charlson Comorbidity Index; NA = not applicable. Reference categories were sedentary for physical activity, male for sex, lowest education level for education, Sweden for country of birth, and no use for each medication variable. Death was treated as a competing event. | | | | | | | | |

**Supplementary Table 3.** Fine–Gray competing-risk regression models with varying adjustments for total stroke, with death treated as a competing event.

|  | Model 1 | | Model 2 | | Model 3 | | Model 4 | |
| --- | --- | --- | --- | --- | --- | --- | --- | --- |
|  | sHR (95% CI) | p-value | sHR (95% CI) | p-value | sHR (95% CI) | p-value | sHR (95% CI) | p-value |
| Physical activity: Light | 0.82 (0.58 - 1.16) | 0.258 | 0.93 (0.65 - 1.33) | 0.685 | 0.91 (0.62 - 1.31) | 0.599 | 0.91 (0.63 - 1.31) | 0.602 |
| Physical activity: Moderate/Vigorous | 0.46 (0.23 - 0.92) | 0.027 | 0.62 (0.31 - 1.25) | 0.178 | 0.60 (0.30 - 1.22) | 0.160 | 0.61 (0.30 - 1.25) | 0.177 |
| Age | NA | NA | 1.04 (1.02 - 1.05) | <0.001 | 1.04 (1.02 - 1.06) | <0.001 | 1.03 (1.02 - 1.05) | <0.001 |
| Sex: Female | NA | NA | 0.96 (0.68 - 1.35) | 0.827 | 0.95 (0.66 - 1.35) | 0.768 | 0.97 (0.68 - 1.39) | 0.874 |
| CCI | NA | NA | NA | NA | 0.81 (0.59 - 1.12) | 0.207 | 0.78 (0.55 - 1.10) | 0.159 |
| Atrial fibrillation | NA | NA | NA | NA | 1.00 (0.65 - 1.55) | 0.987 | 1.02 (0.60 - 1.74) | 0.945 |
| Education: Secondary | NA | NA | NA | NA | 1.03 (0.68 - 1.55) | 0.905 | 1.02 (0.68 - 1.55) | 0.909 |
| Education: Higher | NA | NA | NA | NA | 1.00 (0.63 - 1.61) | 0.989 | 1.00 (0.62 - 1.60) | 0.999 |
| Country of birth: EU without Sweden | NA | NA | NA | NA | 0.98 (0.58 - 1.64) | 0.934 | 0.99 (0.59 - 1.65) | 0.968 |
| Country of birth: Rest of world | NA | NA | NA | NA | 1.08 (0.39 - 2.98) | 0.882 | 1.00 (0.36 - 2.74) | 0.998 |
| Antihypertensive medication | NA | NA | NA | NA | NA | NA | 1.29 (0.86 - 1.95) | 0.220 |
| Lipid-lowering medication | NA | NA | NA | NA | NA | NA | 0.85 (0.55 - 1.30) | 0.456 |
| Antiplatelet medication | NA | NA | NA | NA | NA | NA | 1.62 (1.05 - 2.51) | 0.030 |
| Anticoagulant medication | NA | NA | NA | NA | NA | NA | 1.00 (0.52 - 1.93) | 0.997 |
| **Total cases** | **1610** | | **1610** | | **1568** | | **1568** | |
| **Events** | **137** | | **137** | | **132** | | **132** | |
| **Competing events (death)** | **246** | | **246** | | **217** | | **217** | |
| sHR = subdistribution hazard ratio; CI = confidence interval; CCI = Charlson Comorbidity Index; NA = not applicable. Reference categories were sedentary for physical activity, male for sex, lowest education level for education, Sweden for country of birth, and no use for each medication variable. Death was treated as a competing event. | | | | | | | | |

**Supplementary Table 4.** Fine–Gray competing-risk regression models with varying adjustments for stroke or TIA, with death treated as a competing event.

|  | Model 1 | | Model 2 | | Model 3 | | Model 4 | |
| --- | --- | --- | --- | --- | --- | --- | --- | --- |
|  | sHR (95% CI) | p-value | sHR (95% CI) | p-value | sHR (95% CI) | p-value | sHR (95% CI) | p-value |
| Physical activity: Light | 0.85 (0.68 - 1.05) | 0.121 | 0.91 (0.73 - 1.13) | 0.398 | 0.94 (0.76 - 1.18) | 0.617 | 0.96 (0.77 - 1.19) | 0.687 |
| Physical activity: Moderate/Vigorous | 0.82 (0.58 - 1.15) | 0.251 | 0.98 (0.69 - 1.40) | 0.915 | 1.06 (0.74 - 1.51) | 0.770 | 1.08 (0.75 - 1.55) | 0.689 |
| Age | NA | NA | 1.02 (1.01 - 1.02) | <0.001 | 1.02 (1.01 - 1.03) | 0.002 | 1.01 (1.00 - 1.02) | 0.012 |
| Sex: Female | NA | NA | 1.23 (1.00 - 1.52) | 0.047 | 1.27 (1.03 - 1.57) | 0.028 | 1.30 (1.05 - 1.62) | 0.015 |
| CCI | NA | NA | NA | NA | 0.96 (0.78 - 1.18) | 0.695 | 0.94 (0.76 - 1.17) | 0.601 |
| Atrial fibrillation | NA | NA | NA | NA | 1.10 (0.85 - 1.43) | 0.460 | 1.00 (0.70 - 1.44) | 0.997 |
| Education: Secondary | NA | NA | NA | NA | 0.98 (0.77 - 1.26) | 0.898 | 0.98 (0.77 - 1.25) | 0.875 |
| Education: Higher | NA | NA | NA | NA | 0.87 (0.65 - 1.15) | 0.330 | 0.87 (0.66 - 1.16) | 0.341 |
| Country of birth: EU without Sweden | NA | NA | NA | NA | 1.06 (0.78 - 1.44) | 0.712 | 1.06 (0.78 - 1.45) | 0.696 |
| Country of birth: Rest of world | NA | NA | NA | NA | 1.51 (0.92 - 2.47) | 0.101 | 1.49 (0.92 - 2.43) | 0.108 |
| Antihypertensive medication | NA | NA | NA | NA | NA | NA | 1.03 (0.82 - 1.31) | 0.778 |
| Lipid-lowering medication | NA | NA | NA | NA | NA | NA | 1.08 (0.84 - 1.38) | 0.554 |
| Antiplatelet medication | NA | NA | NA | NA | NA | NA | 1.26 (0.97 - 1.64) | 0.089 |
| Anticoagulant medication | NA | NA | NA | NA | NA | NA | 1.24 (0.80 - 1.92) | 0.333 |
| **Total cases** | **1610** | | **1610** | | **1568** | | **1568** | |
| **Events** | **379** | | **379** | | **364** | | **364** | |
| **Competing events (death)** | **207** | | **207** | | **187** | | **187** | |
| sHR = subdistribution hazard ratio; CI = confidence interval; CCI = Charlson Comorbidity Index; NA = not applicable. Reference categories were sedentary for physical activity, male for sex, lowest education level for education, Sweden for country of birth, and no use for each medication variable. Death was treated as a competing event. | | | | | | | | |

**Supplementary Figure 1.** Distribution of age across pre-TIA physical activity levels. Density curves illustrate the degree of overlap in age distributions between sedentary, light, and moderate/vigorous physical activity groups.

**Supplementary Figure 2.** Exploratory Sankey diagram illustrating physical activity transitions across recurrent Väststroke registrations and subsequent cerebrovascular event types. Each column represents sequential physical activity assessments or event types, and connecting flows illustrate transitions between categories.
